# Cinnamaldehyde inhibits the growth of *Phytophthora capsici* through disturbing metabolic homoeostasis

**DOI:** 10.7717/peerj.11339

**Published:** 2021-04-30

**Authors:** Yinan Wang, Mengke Wang, Min Li, Te Zhao, Lin Zhou

**Affiliations:** 1 Henan Agricultural University, College of Plant Protection, Zhengzhou, Henan, China; 2 Henan Agricultural University, Henan Key Laboratory for Creation and Application of New Pesticides, Zhengzhou, Henan, China; 3 Henan Agricultural University, Henan Research Center of Green Pesticide Engineering and Technology, Zhengzhou, Henan, China

**Keywords:** *Phytophthora capsici* leonian, Cinnamaldehyde, Bioactivity, Quantitative proteomics, Mechanism of action

## Abstract

**Background:**

*Phytophthora capsici* Leonian (*P. capsici*) can cause wilting and roots rotting on pepper and other cash crops. The new fungicide cinnamaldehyde (CA) has high activity against this pathogen. However, its potential mechanism is still unknown.

**Methods:**

In order to gain insights into the mechanism, isobaric tags for relative and absolute quantification (iTRAQ)-based quantitative proteomics was used to analyze *P. capsici* treated with CA. The iTRAQ results were evaluated by parallel reaction monitoring (PRM) analysis and quantitative real-time PCR (qRT-PCR) analysis. Kyoto Encyclopedia of Genes and Genomes (KEGG) enrichment analysis was used to speculate the biochemical pathways that the agent may act on.

**Results:**

The results showed that 1502 differentially expressed proteins were identified, annotated and classified into 209 different terms (like metabolic process, cellular process, single-organism process) based on Gene Ontology (GO) functional enrichment analysis and nine different pathways (glyoxylate and dicarboxylate metabolism, fatty acid metabolism and so on) based on the Kyoto Encyclopedia of Genes and Genomes (KEGG) enrichment analysis. This study suggested that CA disordered fatty acid metabolism, polysaccharide metabolism and leucine metabolism. Based on PRM analysis, five proteins including CAMK/CAMK1 protein kinase, glucan 1,3-beta-glucosidase, 1,3-beta-glucanosyltransferase, methylcrotonoyl-CoA carboxylase subunit alpha and isovaleryl-CoA dehydrogenase were down-regulated in *P. capsici* treated with CA. Furthermore, the qRT-PCR analysis showed that the gene expression level of the interested proteins was consistent with the protein expression level, except for *CAMK/CAMK1 protein kinase*, *acetyl-CoA carboxylase* and *fatty acid synthase subunit alpha*.

**Conclusions:**

CA destroyed the metabolic homoeostasis****of *P. capsici*, which led to cell death. This is the first proteomic analysis of *P. capsici* treated with CA, which may provide an important information for exploring the mechanism of the fungicide CA against *P. capsici*.

## Introduction

*Phytophthora capsici* Leonian, a soilborne oomycete pathogen, can attack many important cash crops, such as solanaceous, legume and most cucurbit hosts (Lamour et al., 2012). The pathogen affects the plants at any development stage and causes damping-off, seedling blight and wilting followed by plant death (Jin et al., 2016). *P. capsici* was described in New Mexico in 1922 for the first time (Leonian, 1922) and had been spread all over the world. It is estimated that this pathogen caused more than one billion dollars loss to global vegetable production each year (Lamour et al., 2012). So far, the diseases caused by *P. capsici* have had damaging impact on the world economy (Chen et al., 2013).

The control of Phytophthora infection is still a persistent agricultural problem. Although crop rotation is an essential basis for disease control, *P. capsici* oospores can survive in the soil for a long time, which obviates its use as antimicrobial strategy (Bishop-Hurley et al., 2002; Hausbeck & Lamour, 2004). Furthermore, the selection of long-lasting and host-resistant varieties may be one of the most effective disease management methods, but currently there are few phytophthora blight-resistant varieties on the market with horticultural traits acceptable to growers (Hausbeck & Lamour, 2004; Ristaino & Johnston, 1999). Therefore, the most commonly implemented measures to control pepper blight are applying chemical fungicides, such as treating the soil with methyl bromide or metalaxyl, or spraying metalaxyl or metalaxyl on plants. Unfortunately, due to the long-term use and highly variable nature of *P. capsici*, some strains had developed resistance or tolerance to these fungicides (Lamour & Hausbeck, 2000; Parra & Ristaino, 2001; Silvar, Merino & Dilv, 2006; Dunn et al., 2010), and there is a limited number of fungicides that can be used against the pathogen (Mei et al., 2015; Pang et al., 2015). So, it is urgent to find more environmentally friendly and safe botanical fungicides.

**Figure 1:**
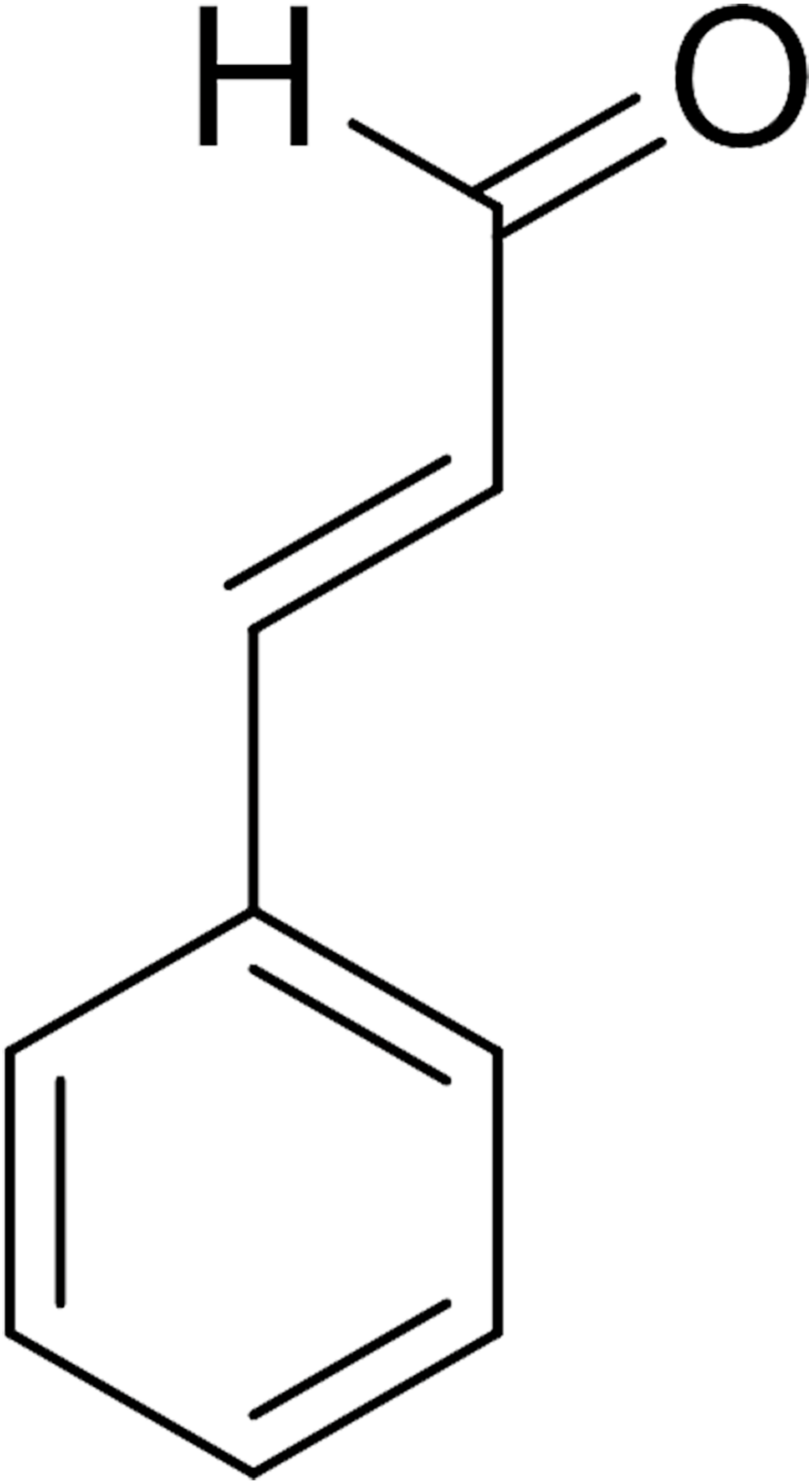
Chemical structure of CA.

Cinnamaldehyde (CA) (Fig. 1) was extracted from the bark and leaves of cinnamon trees, which is the main active ingredients of cinnamon essential oils (Friedman, 2017). CA was used in the food industry due to its antimicrobial activity against bacteria (He et al., 2019), yeast, and filamentous fungi (Sun et al., 2016; Sun et al., 2020). In addition, more interest has been focused on the exploration of CA and its derivatives as promising antifungal drugs (Shreaz et al., 2016). To date, existing experimental evidence shows that CA exerts antimicrobial effects by inhibiting the ATPase activity (Usta et al., 2003), cell wall biosynthesis (OuYang et al., 2019), altering membrane structure and integrity (Huang et al., 2018) and inhibiting cell separation (Kwon, Yu & Park, 2003). Recently, it is speculated that the inhibitory effect of CA on the growth of *P. capsici* was realized by mediating Ca^2+^ efflux in cells (Hu et al., 2013), which provides a significant new idea for the antimicrobial mechanism of CA. However, the target of CA in fungi is not yet clear, which requires more research.

At present, proteomics has been widely used in the field of medicine for target discovery, drug action pattern study and toxicological evaluation of chemical agents (Bantscheff et al., 2007; Ahn & Wang, 2008; Uddin et al., 2019; Xia et al., 2021). However, there are few research reports on the mechanism of action of fungicides by proteomics. Hou et al. (2013) applied proteomic based on two-dimensional gel electrophoresis (2-DE) to analyze the effect of fungicides JS399-19 on protein expression of *Fusarium graminearum*. Mei et al. (2014) also used the 2-DE to study the mechanisms of zoxamide against *Phytophehora cactorum*. The novel antifungal mechanism of benzothiazole against *P. capsici* was explored by proteomic analysis based on iTRAQ combined with transcriptome analysis (Mei et al., 2018). Among these methods, iTRAQ has become an effective tool for exploring the target of fungicides (OuYang, Tao & Zhang, 2018). In this study, to gain more insights into the mechanisms underlying CA toxicity, the expression levels of proteins in *P. capsici* treated with CA were analyzed by iTRAQ. Alterations in protein expression levels were verified using qRT-PCR and PRM (Peterson et al., 2012; Sowers et al., 2015). The proteins related to the inhibition of CA were found, which provided a scientific basis for revealing the inhibition mechanism of CA.

## Material and Methods

### Strain and reagents

*P. capsici* susceptible to conventional treatments was isolated and purified from a stem base of the diseased pepper in Henan Province of China. Single-spore isolate was obtained and cultured for 3 days at 25 °C on V8 agar plates in full darkness for subsequent experiments, in which V8 agar medium was composed of 100 mL filtered V8 juice, 900 mL deionized water, 1.5 g CaCO_3_ and 20 g agar. V8 medium, which was consisted of 100 mL filtered V8 juice, 0.02 g CaCO_3_ and 900 mL deionized water, was prepared for a large number of mycelia of *P. capsici*.

Technical-grade CA was purchased from Sigma and its assay was ≥95%. It was dissolved in acetone and Tween 80 to 3 mg/mL for subsequent experiments, in which acetone and Tween 80 were used as solvents. The medical solution was stored at 4 °C in the dark. qRT-PCR Kit and fluorescence quantitative PCR kit were purchased from TaKaRa [TaKaRa Biotechnology (Dalian) Co., Ltd., China]. Q5 Hot Start High-Fidelity2 × Master Mix was purchased from New England BioLabs (United States).

### *In vitro* effect of CA on mycelial growth of *P. capsici*

Mycelial growth inhibition was used to determine the inhibitory effect of CA on *P. capsici* according to Khan et al.’s method (2011). CA solution was serially diluted by sterile water with 0.4% (vol/vol) acetone and Tween 80 and the desired concentration of CA emulsions were prepared. one mL of CA emulsion was blended with nine mL of V8 agar medium at 40–45 °C to obtain the agar plates amended with CA at 50, 80, 120, 150 and 250 mg/L. The same volume of solvents only was considered as the control.

A fresh mycelial disc (five mm in diameter) was obtained from culturing 3 days *P. capsici* dish and placed on treated plates (90 mm in diameter) after this medium solidification. After six days, each colony diameter was measured by the cross method. And the average value of the colony diameter was used for later data analysis. Each test group was repeated three replicates. According to the formula, the inhibitory rate of CA on the growth of *P. capsici* was calculated. By analyzing the correlation between the logarithm of the CA concentration and the probability value of the *P. capsici* colony growth inhibition rate, the virulence regression equation was obtained. The effective concentration for 50% and 75% inhibition of *P. capsici* mycelial growth (EC_50_, EC_75_) were estimated by the toxicity regression equation (Hou et al., 2020).

\begin{eqnarray*}\text{Inhibition}~ \left( \text{%} \right) =(\mathrm{C}-\mathrm{T})/(\mathrm{C}-5)\times 100 \end{eqnarray*}

Among them, C and T are colony diameter in the control sample and the treated sample respectively.

### Effect of CA on *P. capsici* mycelium morphology

Mycelium morphology was observed by scanning electron microscope. A mycelial disc was placed into Petri dish containing 10 mL of V8 agar medium with a final concentration of 140 mg/L CA. The same volume of solvents only was considered as the control. After 3 days, mycelia were cut into 0.5 cm*0.5 cm pieces and fixed in 2.5% (w/v) glutaraldehyde at room temperature for 24 h. In order to remove the glutaraldehyde solution, the samples were washed with 0.1M PBS (pH = 7.2). The samples were fixed with 1% O_S_O_4_ for 1–2 h. The fixed solution in the sample was washed away by sterile water. The samples were dehydrated by serial rinses with ethanol in water solutions with 30% ethanol for 15 min, 50% ethanol for 20 min, 70%, 80%, 90%, 95%, 100% ethanol for 20 min respectively. After samples were dried and sprayed with gold, *P. capsici* mycelium morphology was observed under Scanning electron microscope.

### Sample preparation for proteomic analysis

*P. capsici* was incubated on V8 agar medium at 25 °C in the dark for 3 days, and then mycelial plugs were obtained at 1/3 of the edge of the colony. Five mycelial plugs were put into Petri dish with 10 mL V8 medium for 3 days, and the culture medium was complemented with CA to produce a final concentration 140 mg/L. Petri dish with no CA was made as control. The mycelia were collected by centrifugation (4 °C, 4 000g, 15 min) and then discarded the supernatant. The mycelia were washed for 3 times with PBS (pH = 7.4) buffer and stored at −80 °C.

### Protein extraction and hydrolysis

Total protein was extracted by acetone/ trichloroacetic acid (TCA) precipitation and SDT lysis method (Méchin, Damerval & Zivy, 2007). Mycelia were pulverized with liquid nitrogen in a mortar and poured into five-time-volume of TCA/ acetone and mixed by vortex. Then they were placed at −20 °C for 5 h. After the supernatant was removed by centrifugation (6000 g for 40 min at 4 °C), the samples were dried in a fume hood to obtain dry powder. The samples were lysed by putting into SDT lysate. The supernatant was collected by centrifugation (14000 g for 40 min) and filtered with a 0.22 M filter. The protein concentration was determined by the BCA protein assay. The total protein concentration was diluted to 100 mM by adding DTT. The mixture was eluted with UA buffer, IAA buffer and dissolution buffer at room temperature and hydrolyzed with 0.1 g/L trypsin buffer at 37 °C for 16 h. Then the peptide fragment was desalted with C18 Cartridge and the lyophilized peptide was dissolved with 40 µL dissolution buffer.

### iTRAQ sample labeling and LC-MS/MS analysis

Six samples (three biological replicates) were labeled with the iTRAQ tags as follows: solvent control (113, 114 and 115), CA treatments (116, 117 and 118). These peptides were incubated and centrifuged in vacuo until dried. The labeled samples were pooled and then graded by using AKTA Purifier 100. The chromatographic column was balanced with loading buffer A (10 mM KH_2_PO_4_ in 25% ACN). The samples were eluted by gradient elution in liquid chromatography. During elution process, the absorbance was detected at 214 nm, and each fraction was separated for one minute. About 30 fractions were obtained and desalted with C18 cartridge after being lyophilized.

Each sample was separated using Nano-HPLC system Easy-nLC. The column was equilibrated with 95% loading buffer A. After the sample was loaded by the autosampler, it was separated by the analytical column. A linear gradient was performed at a velocity of flow of 300 nL/min for 1 h. The loading buffer B (10 mM KH_2_PO_4_ and 500 mM KCl in 25%ACN) was also gradient elution in chromatography.

### Proteomics data processing

This study referred to the uniprot *Phytophthora* protein database. The data was analyzed as Ross et al.’s (2004) described. In short, a normal distribution of iTRAQ ratios was obtained by log2 conversion. And then logarithm, population mean and standard deviation were normalized respectively. Proteins with 1.2-fold change between the two treatments (*P* < 0.05) were identified as differentially expressed proteins, in which were analyzed by GO and KEGG analysis. An average linkage hierarchical clustering analysis was conducted on interested proteins using Multiple Experiment Viewer (MEV v 4.9.0).

### Parallel reaction monitoring (PRM) Analysis

After the same mass protein of each sample was digested by trypsin, the peptide was desalted on the C18 column. The TripleTOF 5600+ LC-MS/MS system (AB SCIEX) was used for targeted MS analysis in PRM. The mass resolution of MS1 scan and MS/MS scan were ∼35000 and ∼15000, respectively. MS data acquisition was carried out in DDA mode to obtain proteins and peptide precursor ions, which were identified by ProteinPilot software. PRM acquisition included a MS1 scan (250 ms), and then a target MS/MS scan, with a period of 1.3–3.3 s. Finally, target proteins were introduced into the software Skyline for processing.

### RNA extraction and qRT-PCR analysis

The mycelia cultured in V8 medium for 3 days (solvent control, CA treatment) were collected in 1.5 mL centrifuge tubes, centrifuged (8000 g, 2 min), and then pulverized with liquid nitrogen in a mortar. Total RNA was extracted with TRIZOL. The RNA was disposed using RNase free DNAse (TaKaRa) at 42 °C for 2 min for removing the DNA contamination. The synthetic cDNA was synthesized from 1 µg of total RNA by a reverse transcription kit. Reaction mixtures (12 µL) contained 6 µL of SYBR Green (TaKaRa), 0.5 μL of forward gene-specific primer, 0.5 µL of reverse gene-specific primer, 1 µL of cDNA, and 4 µL of ddH_2_O. Primers were listed in Table S1. The G3PDH gene was used as the reference.

### Data analysis

All experiments were performed thrice. The data were analyzed using one-way analysis of variance (ANOVA) with PASW Statistics 20.0. Differences with *P* values <0.05 were considered statistically significant.

## Results

### Inhibitory effect of CA on mycelial growth of *P. capsici*

**Figure 2:**
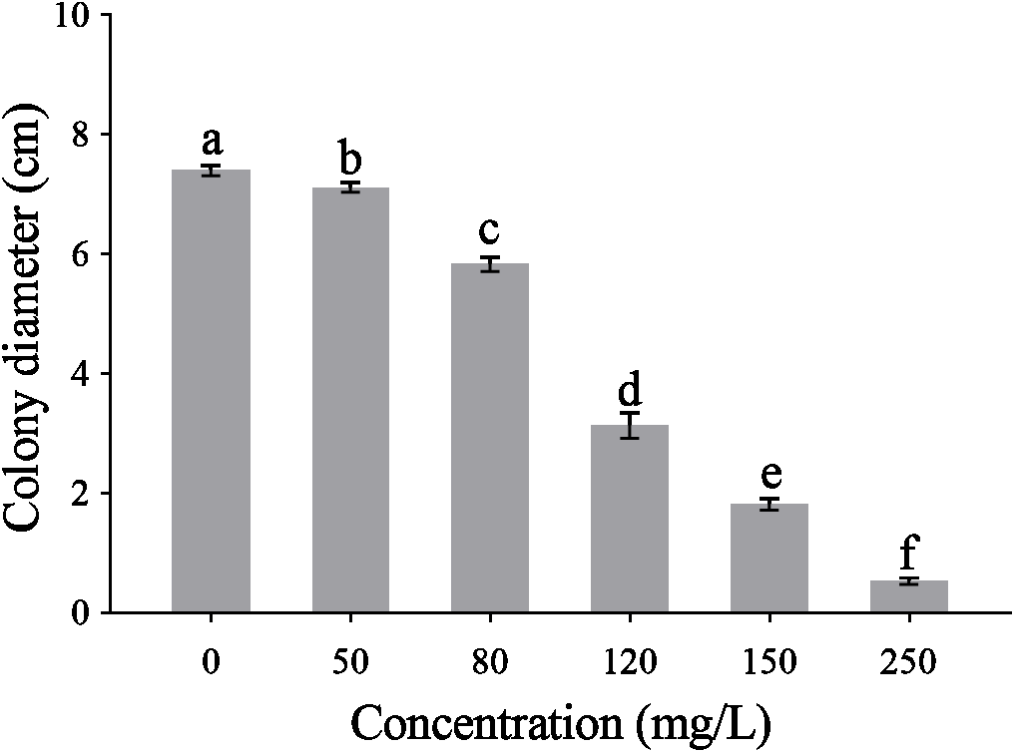
Mycelial growth of *P. capsici* is affected by CA. *P. capsici* was incubated on V8 agar medium at 25 in dark for 6 days and the culture medium were complemented with CA to produce final concentrations 0, 50, 80, 120, 150, 250 mg/L, in which 0 mg/L represented the solvent control. Data presented are the means of the pooled data. Error bars indicate the SDs of the means (*n* = 3).

The CA obviously effect the mycelial radical elongation of *P. capsici* (Fig. 2). CA of higher concentration showed stronger inhibitory effect against *P. capsica*. The toxic regression equation of the CA for *P. capsici* was *Y* =  − 6.1485 + 5.5080*X*. Moreover, the EC_50_ and EC_75_ values of CA against *P. capsici* were 105.69 and 140.12 mg/L respectively.

### Effect of CA on the mycelial morphology

**Figure 3:**
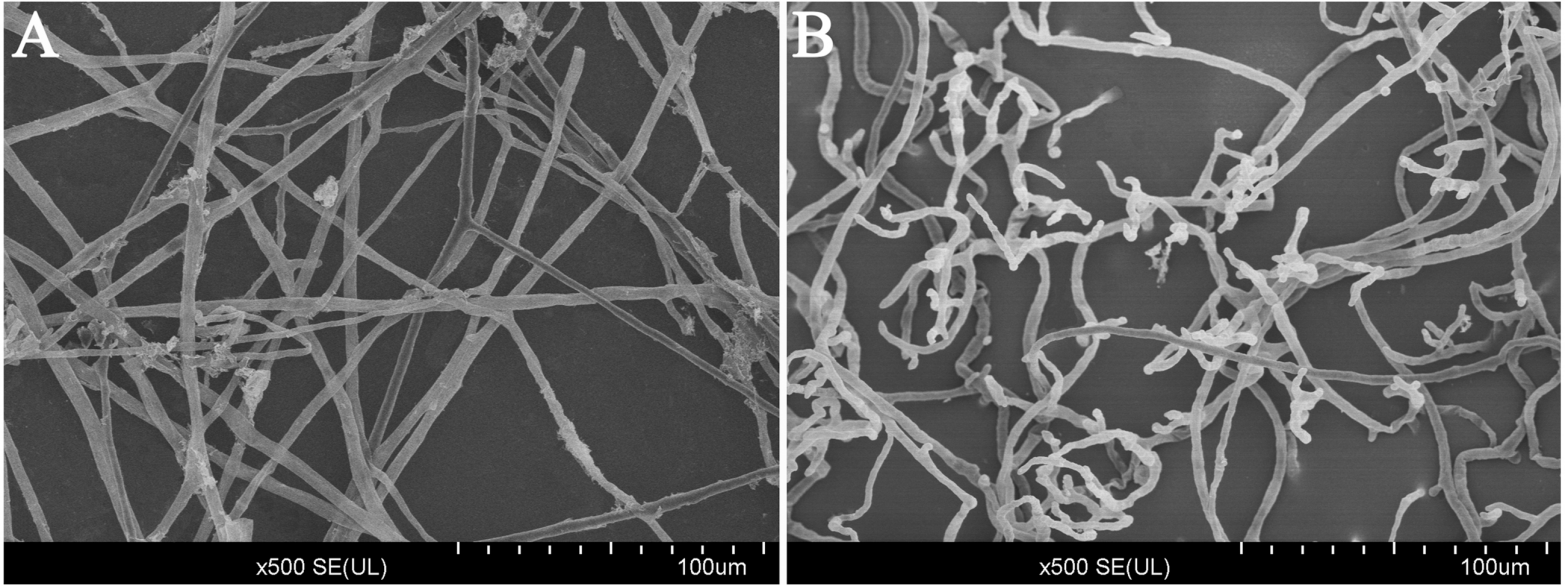
Scanning electron microscope images (500×) of the morphology of *P. capsici* with and without treatment with CA. (A) Mycelia of untreated *P. capsici* (solvent control) cultured for 3 days; (B) mycelia of *P. capsici* treated with CA at 140 mg/L for 3 days.

After treated with CA (140 mg/L) for 3 days in V8 agar medium, the results of scanning electron microscopy (SEM) observations revealed that the mycelium growth of *P. capsici* was significantly limited (Fig. 3). In addition, the mycelium was distorted and shriveled. The embranchment increased. While, the mycelium of the control group was stretched, uniform and full. And the surface of mycelium was smooth and the diameter of mycelium was constant (Fig. S1). Since the CA of EC_75_ concentration has obvious inhibition on the mycelium growth of *P. capsici*, the EC_75_ value was selected as the test concentration in this study.

### Overview of the quantitative proteomics analysis of *P. capsici* treated with CA

**Figure 4:**
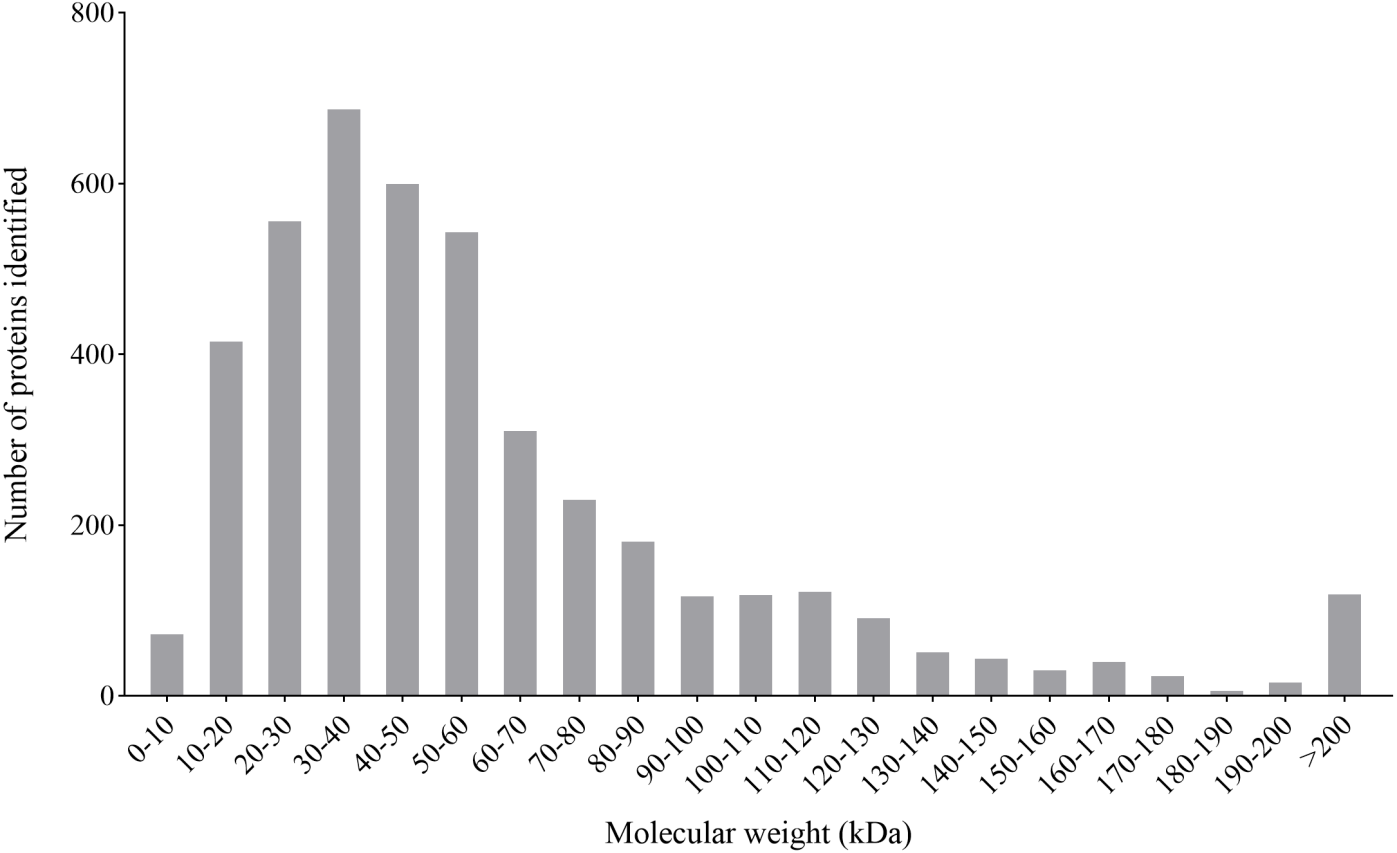
Molecular weight distribution of the proteins identified from the iTRAQ analysis of *P. capsici* challenged by CA.

By the iTARQ-LC-MS/MS analysis, a total of 4370 proteins were identified in *P. capsici* treated with CA (140 mg/L) for 3 days. Most proteins have a molecular weight between 10–70 kDa, in which the number of proteins with molecular weight between 30–40 kDa was in the largest proportion (Fig. 4).

### Effect of CA on protein levels

With the cut-off values *P* ≤ 0.05 and fold changes >1.2 or ≤0.83, proteins between the two samples were considered differentially expressed. Compared CA treatment with control group, a total of 1502 differentially expressed proteins were identified of which 647 proteins were up-regulated and 855 down- regulated based on the cut-off value (Table S2).

### *P. capsici* proteins related to the mode of action of CA

**Figure 5:**
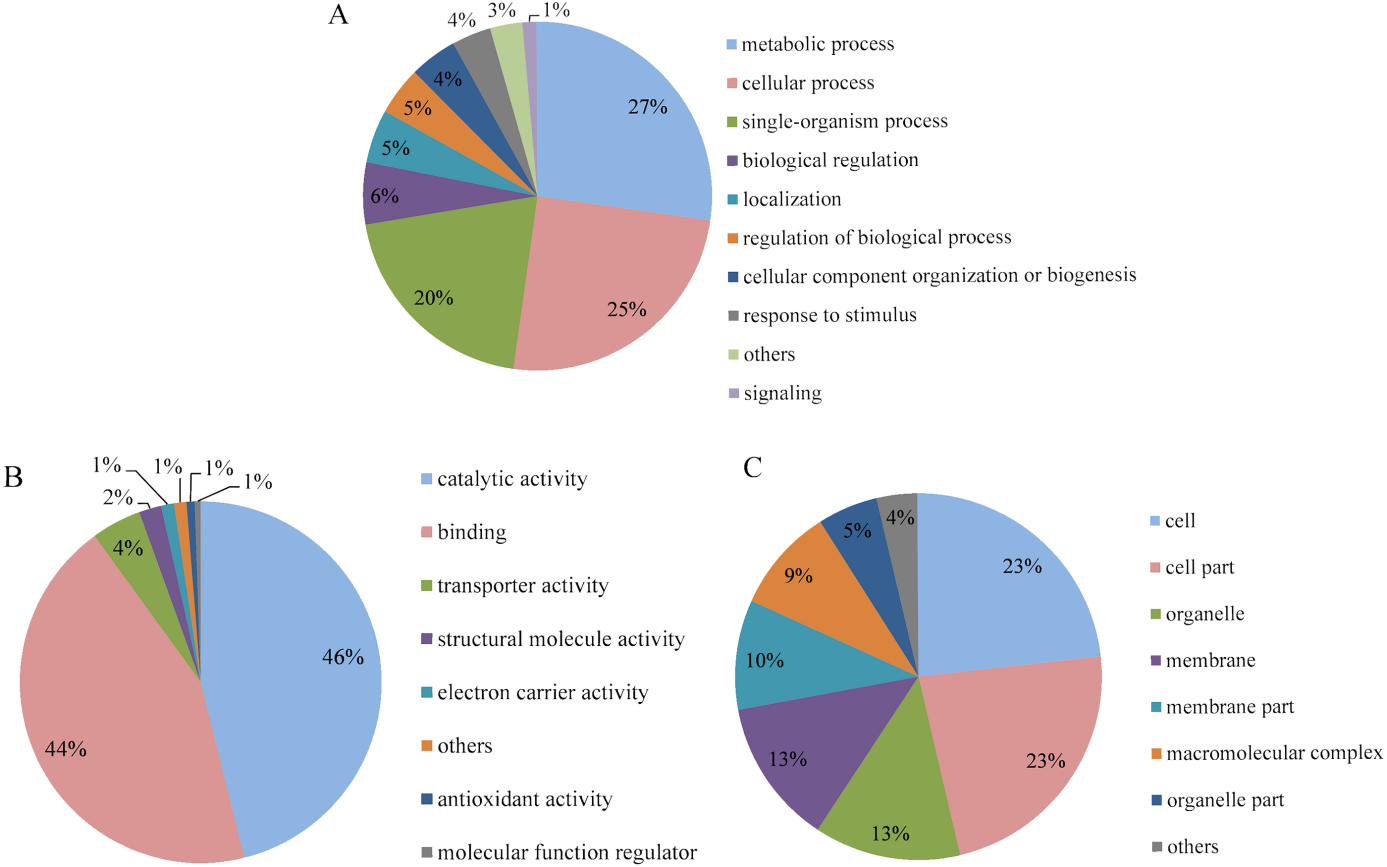
GO analysis of differentially expressed proteins identified on CA-treated *P. capsici* (*P* value < 0.05). (A) Biological process. (B) Molecular function. (C) Cellular component.

Go analysis was performed for level 2, and the differentially expressed proteins were categorized according to their biological process, molecular function and cellular component. The biological process annotation revealed that the differentially expressed proteins of *P. capsici* treated with CA were involved in response to metabolic process (27%), cellular process (25%) and single-organism process (20%) (Fig. 5A). In the molecular function, as shown in Fig. 5B, the number of differentially expressed proteins involved in catalytic activity and binding, which accounted for 46% and 44%, respectively. In the category of cell component (Fig. 5C), the differentially expressed proteins involved in cell and cell part were the most, accounting for 23% respectively. So, CA mainly caused significant changes in protein expression related to metabolism, cell process, catalytic activity, protein binding and cell and cell separation in *P. capsici*, indicating that these differential proteins may be involved in the main biochemical pathway of CA acting on the target pathogen.

**Figure 6:**
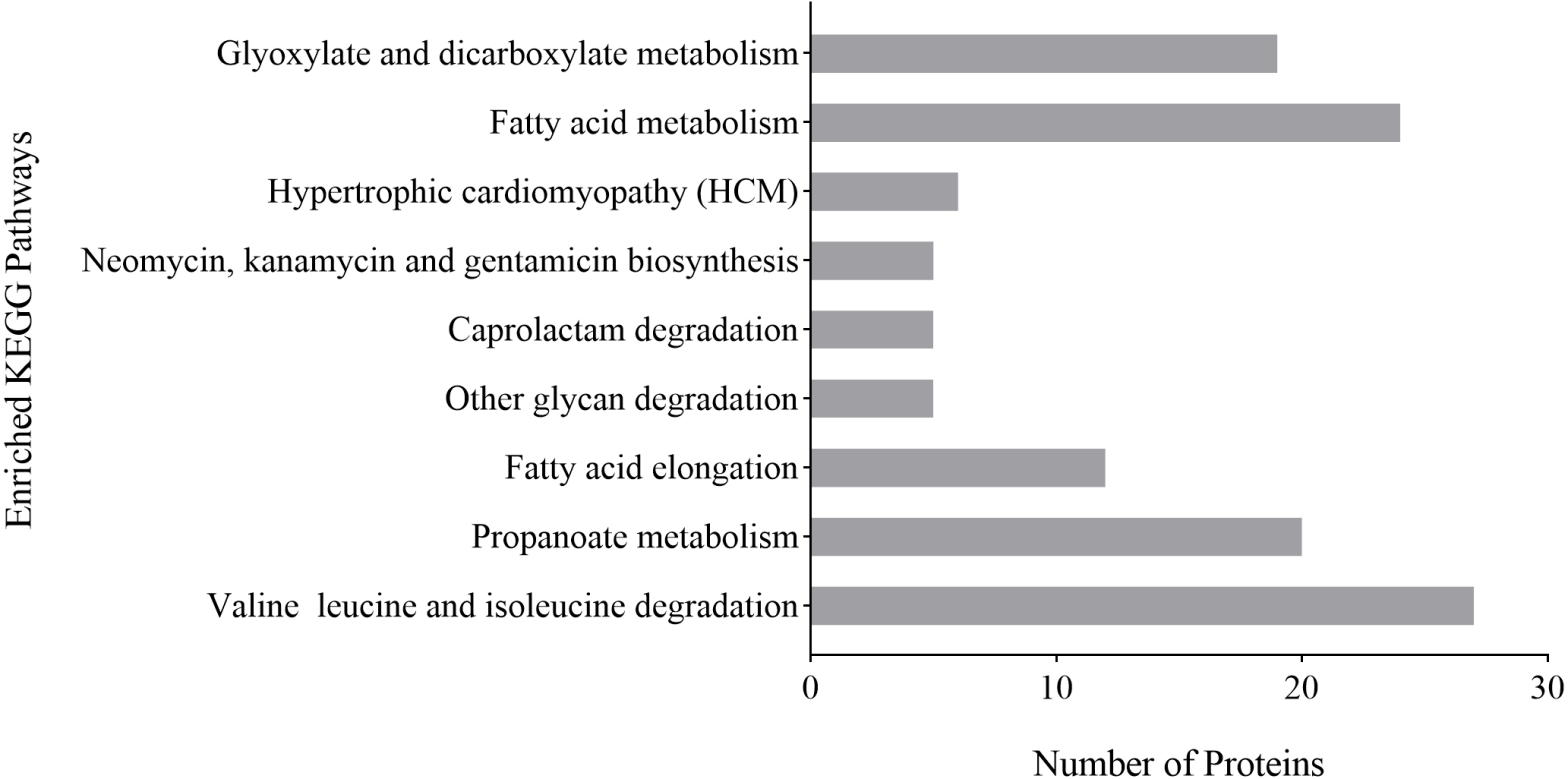
Analysis of KEGG enrichment of differential proteins identified on CA-treated *P. capsici* (*P* value < 0.05).

KEGG pathway analysis was used to analyze possible links between a protein and biochemical pathways. The differentially expressed proteins of *P. capsici* treated with CA were related to degradation pathways, including caprolactam degradation, other glycan degradation and valine, leucine and isoleucine degradation; associated with fatty acid pathways, including fatty acid metabolism and fatty acid elongation and involved with other pathways, including glyoxylate and dicarboxylate metabolism, hypertrophic cardiomyopathy, neomycin, kanamycin and gentamicin biosynthesis, propanoate metabolism (Fig. 6). These results indicated that fatty acid metabolism and the degradation of valine, leucine and isoleucine might play an important role in the inhibition of *P. capsica* by CA.

### Functional classification of interested differentially expressed proteins

**Figure 7:**
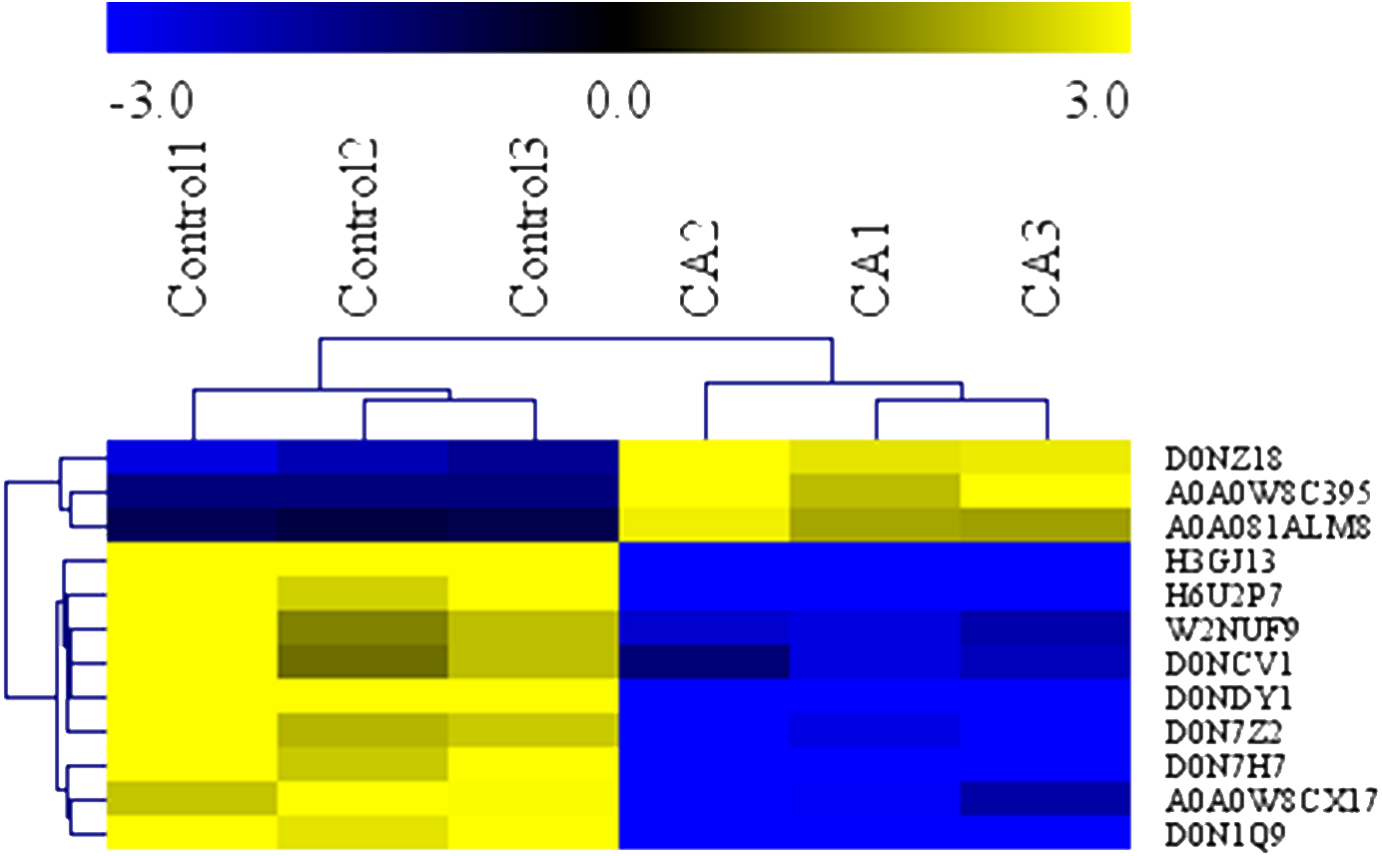
Heatmap of protein expression levels in in solvent control and CA treatment. Blue represents the down-regulated proteins (fold change ≤ 0.83 and *P* Value < 0.05), and yellow represents the up-regulated proteins (fold change > 1.2 and *P* value < 0.05).

To determine the antimicrobial action of CA against *P. capsici*, we used the GO terms data and KEGG data to analyze the biochemical pathways involved in differentially expressed proteins. Analysis demonstrated that the 12 interested proteins involved in fatty acid metabolism (3 down-regulated, 3 up-regulated), polysaccharide metabolic (4 down-regulated) and leucine metabolism (2 down-regulated) were also significantly altered (Table 1). As shown in Fig. 7, the heat map of hierarchical clustering verified the rationality and accuracy of the selected differentially expressed proteins.

### PRM verification of interested proteins

PRM technology based on mass spectrometry was used to quantitatively verify protein. Five interested proteins including glucan 1,3-beta-glucosidase, 1,3-beta-glucanosyltransferase, CAMK/CAMK1 protein kinase, methylcrotonoyl-CoA carboxylase subunit alpha and isovaleryl-CoA dehydrogenase were selected for initial validation. The PRM results showed that the five proteins of *P. capsici* treated with CA were down-regulated (Table 2), which was consistent with the results of proteomics analysis.

### Quantitative of qRT-PCR analysis

**Figure 8:**
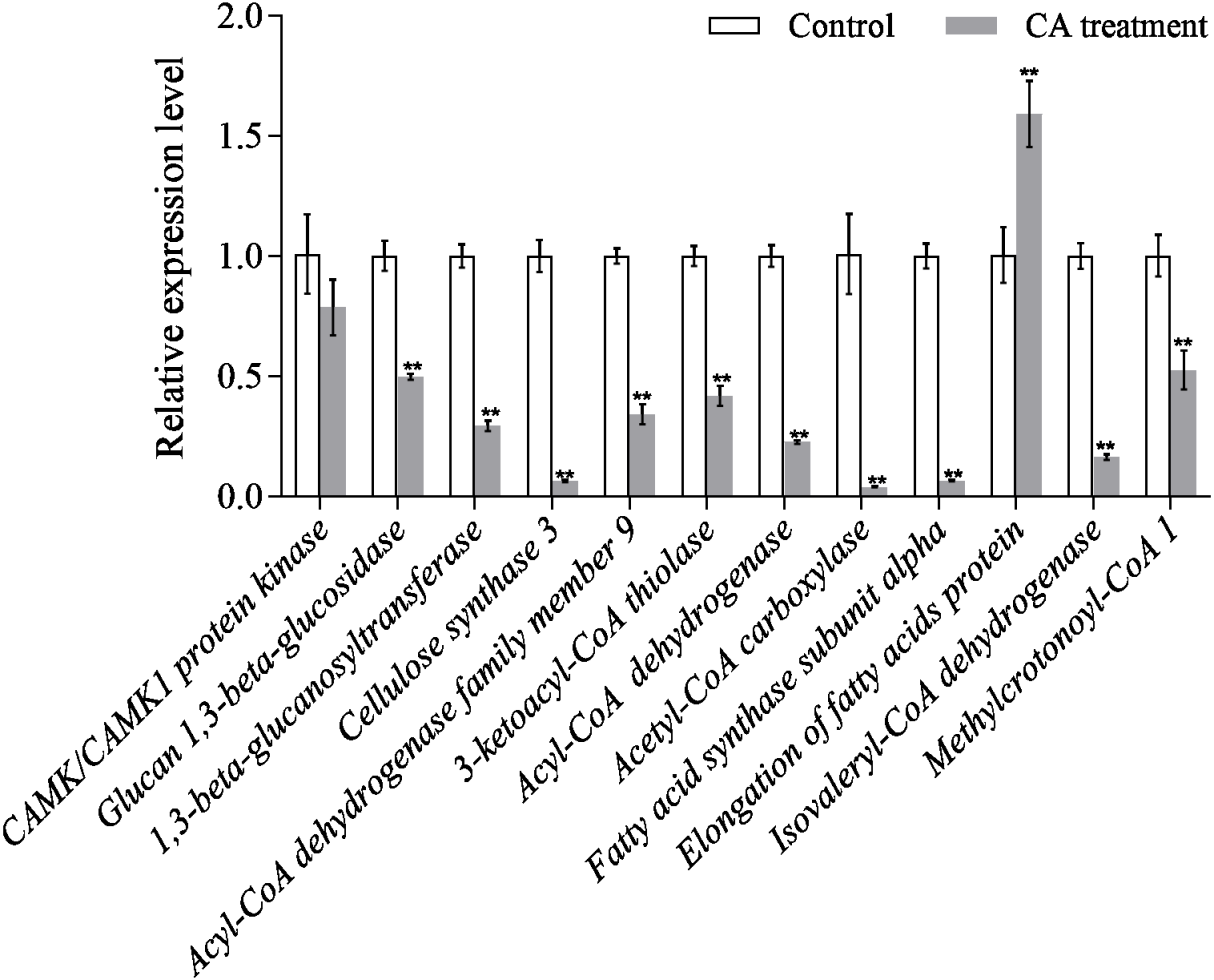
Relative expression of mRNAs from twelve genes as determined by qRT-PCR. Bars represent the means ± SD (^∗^*P* < 0.05, and ^∗∗^*P* < 0.01 with compared to CA treatment).

The results of qRT-PCR showed that the gene expression level of the interested proteins was consistent with the protein expression level, except for *CAMK/CAMK1 protein kinase*, *acetyl-CoA carboxylase* and *fatty acid synthase subunit alpha* (Fig. 8). Moreover, the protein expression of cellulose synthase 3 decreased 1.43-fold while its RNA expression decreased 15.73-fold. The discrepancy between protein expression and RNA expression may be caused by the time difference between transcription and translation.

**Table 1:** Functional classification of interested differentially expressed proteins of *P. capsici* treated with CA.

Accession	Description	Regulation level / fold change
Fatty acid metabolism		
D0NDY1	Acyl-CoA dehydrogenase family member 9	Down/0.76
D0N7H7	3-ketoacyl-CoA thiolase	Down/0.74
D0N7Z2	Acyl-CoA dehydrogenase, putative	Down/0.62
D0NZ18	Acetyl-CoA carboxylase, putative	Up/1.69
A0A0W8C395	Fatty acid synthase subunit alpha	Up/2.16
A0A081ALM8	Elongation of fatty acids protein	Up/3.07
Polysaccharide metabolic		
W2NUF9	CAMK/CAMK1 protein kinase	Down/0.53
D0NCV1	Glucan 1,3-beta-glucosidase, putative	Down/0.48
H3GJ13	1,3-beta-glucanosyltransferase	Down/0.81
H6U2P7	Cellulose synthase 3	Down/0.70
Leucine metabolism		
A0A0W8CX17	Isovaleryl-CoA dehydrogenase	Down/0.63
D0N1Q9	Methylcrotonoyl-CoA carboxylase 1	Down/0.77

## Discussion

**Figure 9:**
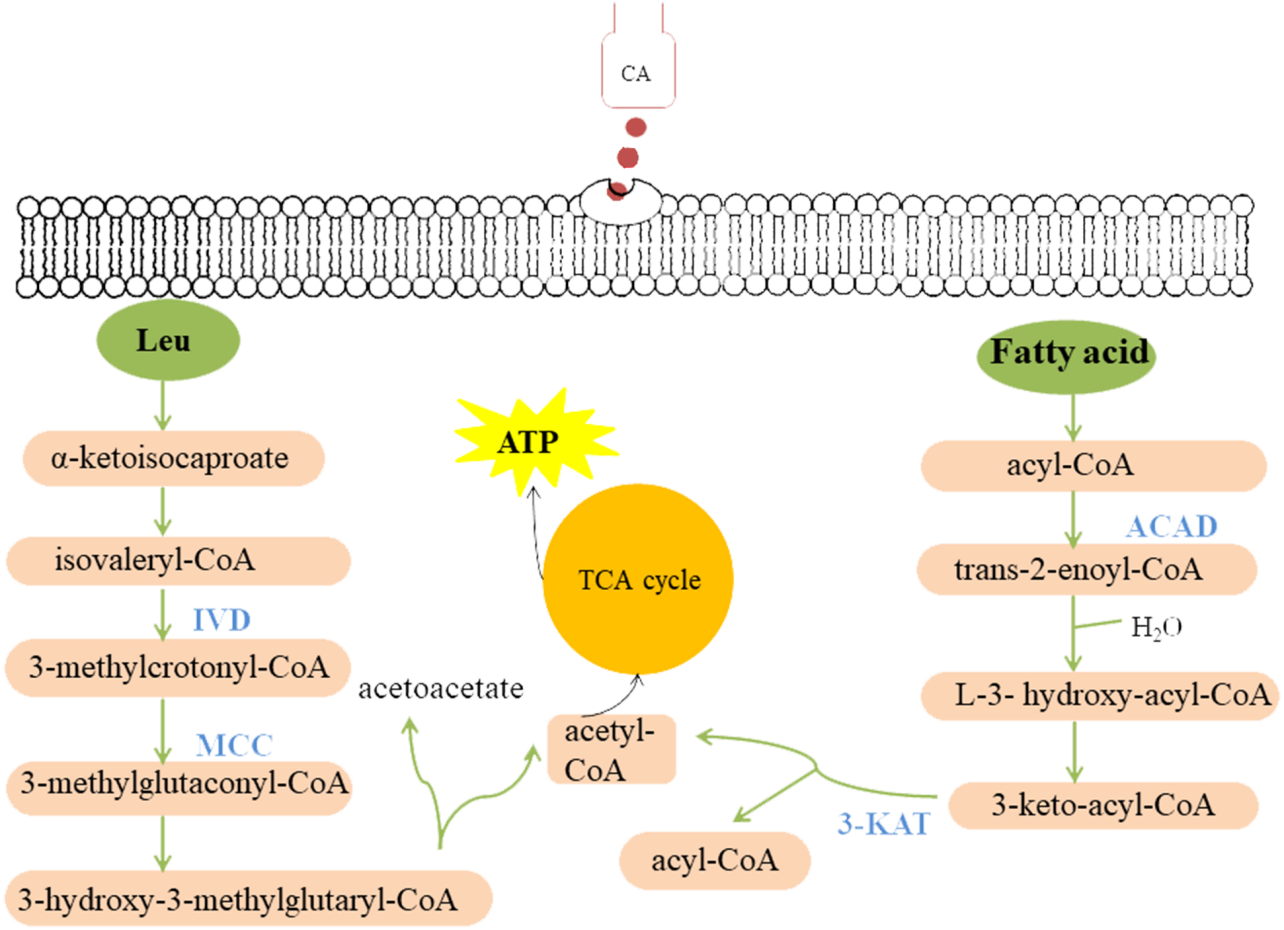
Model summarized antimicrobial effects of CA on *P. capsici*. Each green ellipse represents a pathway, each pink box represents a substance, and the orange circle represents the TCA cycle. Blue proteins indicate down-regulated. IVD, isovaleryl-CoA dehydrogenase; MCC, methylcrotonyl-CoA carboxylase subunit alpha; 3-KAT, 3-Ketoacyl-CoA thiolase; ACAD, Acyl-CoA dehydrogenase.

There are few reports on the mechanism of action of CA on fungi and oomycetes. OuYang et al. (2019) found that after CA treatment, the cell wall of *Geotrichum citri-aurantii* was dissolved and the chitin content was reduced, therefore they concluded that CA could inhibit the mycelial growth by destroying the integrity of cell wall. Shreaz et al. (2011) found that plasma membrane ATPase activity of *C. albicans* treated with CA was inhibited, which led to the increase of intracellular H^+^ concentration and cell death. Hu et al. (2013) determined the free Ca^2+^ concentration in the zoospores of *P. capsici* before and after CA treatment, and found that CA inhibits mycelial growth by causing Ca^2+^ efflux. In this study, based on the strong inhibition of CA on the mycelial growth of *P. capsici*, a total of 1502 differentially expressed proteins were identified by quantitative proteomics. These differentially expressed proteins were mostly involved in fatty acid metabolism, polysaccharide metabolism, and leucine metabolism (Table 1). It has been reported that glucose, fatty acids and amino acids are the three substrates for organisms to maintain metabolic homoeostasis (Sander & Ronald, 2010). Therefore, CA may break the metabolic balance of the oomycete, causing to inhibit pathogen growth (Fig. 9).

**Table 2:** Interested proteomics data validation by PRM.

Accession	Description	Regulation level / fold change
Polysaccharide metabolic		
W2NUF9	CAMK/CAMK1 protein kinase	Down/0.319
D0NCV1	Glucan 1,3-beta-glucosidase	Down/0.408
H3GJ13	1,3-beta-glucanosyltransferase	Down/0.491
Leucine metabolism		
D0N1Q9	Methylcrotonoyl-CoA carboxylase subunit alpha, putative	Down/0.453
A0A0W8CX17	Isovaleryl-CoA dehydrogenase	Down/0.345
Internal reference protein		
A0A0W8DRZ2	40S ribosomal protein S13	
Q2M3Z6	Ribosomal protein L23	

Fatty acid metabolism is closely related to fungal growth (Calvo, Gardner & Keller, 2001). In this study, both Acyl-CoA dehydrogenase (ACAD) and 3-Ketoacyl-CoA thiolase (3-KAT) were down-regulated after CA treatment of *P. capsici* by proteomics analysis. The results of qRT-PCR showed that *3-ketoacyl-CoA thiolase* and *acyl-CoA dehydrogenase* were also down-regulated by CA treatment Fig. 8. And the down-regulated ACAD and 3-KAT slowed down the metabolism of fatty acids, which might lead to the increase of fatty acid content (Wang et al., 2018a; Wang et al., 2018b). In addition, the up-regulated acetyl CoA carboxylase (ACC), the up-regulated fatty acid synthase subunit alpha and the up-regulated elongation of fatty acids protein also promoted the synthesis of fatty acids. Previous studies also found that *Penicillium expansum* treatment with CA changed the expression of proteins related to fatty acid metabolism (Wang et al., 2018a; Wang et al., 2018b).

Cellulose, an important component of the cell wall of oomycetes (Bartnicki-Garcia, 1968), is mainly synthesized by cellulose synthetase. Down-regulated cellulose synthetase and glucan 1,3- beta-glucosidase (Igarashi et al., 2003) might disrupt cell wall metabolism in fungi. Shreaz et al. (2013) observed that the cell wall of *Candida* treated with CA was damaged by transmission electron microscope. CAMK/CAMK1 protein kinase 1 participates in the regulation of production of conidiation and mycelial growth (Liu et al., 2010). 1,3-beta-glucanosyltransferase, the Gel/Gas/Phr family of proteins, contributes a lot in the modification of fungal cell wall structure and it is considered to be related to the mycelial embranchment (Mouyna, Hartl & Latgé, 2013). Previous studies found (Kamei et al., 2013) that the deletion of the *gel-3* in *Neurospora crassa* caused that the mycelium grew slowly compared with wild strain. Down-regulated CAMK/CAMK1 protein and 1,3-beta-glucanosyltransferase might result in changes in mycelia morphology. Moreover, the scanning electron microscope observed that the growth of mycelium was inhibited, and the mycelial embranchment increased. In this study, cellulose synthase 3, glucan 1,3-beta-glucosidase, CAMK/CAMK1 protein kinase and 1,3-beta-glucanosyltransferase were down-regulated in *P. capsici* treated with CA, which not only changed the cell morphology and mycelial morphology of *P. capsici*, but also disturbed the polysaccharide metabolism.

In this study, isovaleryl-CoA dehydrogenase (IVD) and methylcrotonyl-CoA carboxylase subunit alpha (MCC) are the key enzymes of mitochondrial leucine metabolism (Mentzen et al., 2008), in which IVD catalyzes the conversion of isovaleryl-CoA to 3-methylcrotonyl-CoA (Mohsen & Vockley, 1995). Down-regulated IVD might reduce the production of 3-methylcrotonyl-CoA, which is the catalytic substrate of MCC to generate 3-methylglutaconyl-CoA. The MCC can prevent the store of toxic substances and provide energy to the cell (Tomassetti et al., 2018). Rodríguez et al. (2004) indicated that one or more of the metabolites accumulating in the MCC-deficient strains are toxic to the cells. Proteomic and PRM data all demonstrated that MCC and IVD were down-regulated after treated with CA, which might lead to the accumulation of intracellular metabolites, breaking the normal cell environment and causing cell death.

## Conclusion

In conclusion, CA has good antimicrobial activity against *P. capsici*. CA disturbs fatty acid metabolism, polysaccharide metabolism and leucine metabolism of *P. capsici*, which lead to cell dysfunction or even cell death. Therefore, CA is expected to become a new fungicide for the control of pepper blight.

##  Supplemental Information

10.7717/peerj.11339/supp-1 Supplemental Information 1 Primers for qRT-PCR analysis

10.7717/peerj.11339/supp-2 Supplemental Information 2 Differentially expressed proteins identified by proteomics

10.7717/peerj.11339/supp-3 Supplemental Information 3 Scanning electron microscope images (4.5 k ×) of the morphology of *P. capsici* with and without treatment with CA (C) Mycelium of untreated culture (solvent control) treated for 3 days; (D) mycelium of*P. capsici* treated with CA at 140 mg/L for 3 days.

10.7717/peerj.11339/supp-4 Supplemental Information 4 Molecular weight distribution of the proteins identified from the iTRAQ analysis of *P. capsici* challenged by CA

10.7717/peerj.11339/supp-5 Supplemental Information 5 GO analysis of differentially expressed proteins

10.7717/peerj.11339/supp-6 Supplemental Information 6 Analysis of KEGG enrichment of differential proteins

10.7717/peerj.11339/supp-7 Supplemental Information 7 Relative expression of mRNAs from genes as determined by qRT-PCR

## Additional Information and Declarations

### Data Availability

The following information was supplied regarding data availability: The raw measurements are available in the Supplemental Files.

## References

[ref-1] Ahn NG, Wang AH (2008). Proteomics and genomics: perspectives on drug and target discovery. Current Opinion in Chemical Biology.

[ref-2] Bantscheff M, Eberhard D, Abraham Y, Bastuck S, Boesche M, Hobson S, Mathieson T, Perrin J, Raida M, Rau C, Reader V, Sweetman G, Bauer A, Bouwmeester T, Hopf C (2007). Quantitative chemical proteomics reveals mechanisms of action of clinical ABL kinase inhibitors. Nature Biotechnology.

[ref-3] Bartnicki-Garcia S (1968). Cell wall chemistry, morphogenesis, and taxonomy of fungi. Annual Review of Microbiology.

[ref-4] Bishop-Hurley SL, Mounter SA, Laskey J, Morris RO, Elder J, Roop P, Rouse C, Schmidt FJ, English JT (2002). Phage-displayed peptides as developmental xgonists for *Phytophthora capsici* zoospores. Applied and Environmental Micaobioloyg.

[ref-5] Calvo AM, Gardner HW, Keller NP (2001). Genetic connection between fatty acid metabolism and sporulation in *Aspergillus nidulans*. Journal of Biological Chemistry.

[ref-6] Chen XR, Xin YP, Li YP, Tong YH, Xu JY (2013). RNA-Seq reveals infection-related gene expression changes in *Phytophthora capsici*. PLOS ONE.

[ref-7] Dunn AR, Milgroom MG, Meitz JC, McLeod A, Fry WE, McGrath MT, Dillard HR, Smart CD (2010). Population structure and resistance to mefenoxam of *Phytophthora capsici* in New York State. Plant Disease.

[ref-8] Friedman M (2017). Chemistry, antimicrobial mechanisms, and antibiotic activities of Cinnamaldehyde against pathogenic bacteria in animal feeds and human foods. Journal of Agricultural and Food Chemistry.

[ref-9] Hausbeck MK, Lamour KH (2004). *Phytophthora capsici* on vegetable crops: research progress and management challenges. Plant Disease.

[ref-10] He ZY, Huang ZW, Jiang W, Zhou W (2019). Antimicrobial Activity of Cinnamaldehyde on *Streptococcus mutans* Biofilms. Frontiers in Microbiology.

[ref-11] Hou HY, Zhang XY, Zhao T, Zhou L (2020). Effects of *Origanum vulgare* essential oil and its two main components, carvacrol and thymol, on the plant pathogen *Botrytis cinerea*. PeerJ.

[ref-12] Hou YP, Zheng ZT, Xu S, Chen CJ, Zhou MG (2013). Proteomic analysis of *Fusarium graminearum* treated by JS399-19. Pesticide Biochemical and Physiology.

[ref-13] Hu LB, Wang DD, Liu L, Chen J, Xue YF, Shi ZQ (2013). Ca^2+^ efflux is involved in cinnamaldehyde-induced growth inhibition of *Phytophthora capsici*. PLOS ONE.

[ref-14] Huang F, Kong J, Ju J, Zhang Y, Guo Y, Cheng Y, Qian H, Xie Y, Yao W (2018). Membrane damage mechanism contributes to inhibition of trans-cinnamaldehyde on *Penicillium italicum* using Surface-Enhanced Raman Spectroscopy (SERS). Scientific Reports.

[ref-15] Igarashi K, Tani T, Kawai R, Samejima M (2003). Family 3 beta-glucosidase from cellulose-degrading culture of the white-rot fungus *Phanerochaete chrysosporium* is a glucan 1, 3-beta-glucosidase. Journal of Bioscience & Bioengineering.

[ref-16] Jin JH, Zhang HX, Tan JY, Yan MJ, Li DW, Khan A, Gong ZH (2016). A New ethylene-responsive factor CaPTI1 gene of pepper (*Capsicum annuum* L.) involved in the regulation of defense response to *Phytophthora capsici*. Frontiers in Plant Science.

[ref-17] Kamei M, Yamashita K, Takahashi M, Fukumori F, Ichiishi A, Fujimura M (2013). Deletion and expression analysis of beta-(1, 3)-glucanosyltransferase genes in *Neurospora crassa*. Fungal Genetics & Biology.

[ref-18] Khan MA, Cheng ZH, Xiao XM, Khan AR, Muhammad ASS (2011). Ultrastructural studies of the inhibition effect against *Phytophthora capsici* of root exudates collected from two garlic cultivars along with their qualitative analysis. Crop Protection.

[ref-19] Kwon JA, Yu CB, Park HD (2003). Bacteriocidal effects and inhibition of cell separation of cinnamic aldehyde on *Bacillus cereus*. Letters in Applied Microbiology.

[ref-20] Lamour KH, Hausbeck MK (2000). Mefenoxam insensitivity and the sexual stage of *Phytophthora capsici* in Michigan cucurbit fields. Phytopathology.

[ref-21] Lamour KH, Stam R, Jupe J, Huitema E (2012). The oomycete broad-host-range pathogen *Phytophthora capsici*. Molecular Plant Pathology.

[ref-22] Leonian LH (1922). Stem and fruit blight of peppers caused by *Phytophthora capsici* sp. nov. Phytopathology.

[ref-23] Liu XH, Lu JP, Dong B, Gu Y, Lin FC (2010). Disruption of *MoCMK1*, encoding a putative calcium/calmodulin-dependent kinase, in *Magnaporthe oryzae*. Microbiological Research.

[ref-24] Mei XY, Liu YX, Huang HC, Du F, Huang LL, Wu JQ, Li YW, Zhu SS, Yang M (2018). Benzothiazole inhibits the growth of *Phytophthora capsici* through inducing apoptosis and suppressing stress responses and metabolic detoxification. Pesticide Biochemistry and Physiology.

[ref-25] Mei XY, Yang M, Ding XP, Bi Y, Chen L, Deng WP, Dong YM, Su Y, He XH, Zhu SS, Liu XL (2014). Proteomic analysis of zoxamide-induced changes in *Phytophthora cactorum*. Pesticide Biochemistry and Physiology.

[ref-26] Mei XY, Yang M, Jiang BB, Ding XP, Deng WP, Dong YM, Chen L, Liu XL, Zhu SS (2015). Proteomic analysis on zoxamide-induced sensitivity changes in *Phytophthora cactorum*. Pesticide Biochemical Physiology.

[ref-27] Mentzen WI, Peng J, Ransom N, Nikolau BJ, Wurtele ES (2008). Articulation of three core metabolic processes in arabidopsis: fatty acid biosynthesis, leucine catabolism and starch metabolism. BMC Plant Biology.

[ref-28] Mohsen AW, Vockley J (1995). High-level expression of an altered cDNA encoding human isovaleryl-CoA dehydrogenase in *Escherichia coli*. Gene.

[ref-29] Mouyna I, Hartl L, Latgé JP (2013). β-1, 3-glucan modifying enzymes in *Aspergillus fumigatus*. Frontiers in Microbiology.

[ref-30] OuYang QL, Duan XF, Li L, Tao NG (2019). Cinnamaldehyde exerts its antifungal activity by disrupting the cell wall integrity of *Geotrichum citri-aurantii*. Frontiers in Microbiology.

[ref-31] OuYang QL, Tao NG, Zhang ML (2018). A damaged oxidative posphorylation mechanism is involved in the antifungal activity of citral against *Penicillium digitatum*. Frontiers in Microbiology.

[ref-32] Pang ZL, Chen L, Miao JQ, Wang ZW, Bulone V, Liu XL (2015). Proteomic profile of the plant-pathogenic oomycete *Phytophthora capsici* in response to the fungicide pyrimorph. Proteomics.

[ref-33] Parra G, Ristaino JB (2001). Resistance to mefenoxam and metalaxyl among field isolates of *Phytophthora capsica* causing Phytophthora blight of bell pepper. Plant Disease.

[ref-34] Peterson AC, Russell JD, Bailey DJ, Westphall MS, Coon JJ (2012). Parallel reaction monitoring for high resolution and high mass accuracy quantitative, targeted proteomics. Molecular & Cellular Proteomics.

[ref-35] Ristaino JB, Johnston SA (1999). Ecologically based approaches to management of Phytophthora blight on bell pepper. Plant Disease.

[ref-36] Rodríguez JM, Ruízsala-Sala P, Ugarte M, Penalva MA (2004). Fungal metabolic model for 3-methylcrotonyl-CoA carboxylase deficiency. The Journal of Biological Chemistry.

[ref-37] Ross PL, Huang YN, Marchese J, Williamson B, Parker K, Hattan S, Khainovski K, Pillai S, Dey S, Daniels S, Purkayastha S, Juhasz P, Martin S, Bartlet-Jones M, He F, Jacobson A, Pappin DJ (2004). Multiplexed protein quantitation in *Saccharomyces cerevisiae* using amine-reactive isobaric tagging reagents. Molecular & Cellular Proteomics.

[ref-38] Sander MH, Ronald JAW (2010). A general introduction to the biochemistry of mitochondrial fatty acid β-oxidation. Journal of Inherited Metabolic Disease.

[ref-39] Shreaz S, Bhatia R, Khan N, Muralidhar S, Basir SF, Manzoor N, Khan LA (2011). Spice oil cinnamaldehyde exhibits potent anticandidal activity against fluconazole resistant clinical isolates. Fitoterapia.

[ref-40] Shreaz S, Bhatia R, Khan N, Muralidhar S, Manzoor N, Khan LA (2013). Influences of cinnamic aldehydes on H^+^ extrusion activity and ultrastructure of *Candida*. Journal of Medical Microbiology.

[ref-41] Shreaz S, Wani WA, Behbehani JM, Raja V, Irshad M, Karched M, Ali I, Siddiqi WA, Hun LT (2016). Cinnamaldehyde and its derivatives, a novel class of antifungal agents. Fitoterapia.

[ref-42] Silvar C, Merino F, Dilv J (2006). Diversity of *Phytophthora capsici* in Northwest Spain: Analysis of virulence, metalaxyl response, and molecular characterization. Plant Disease.

[ref-43] Sowers JL, Mirfattah B, Xu P, Tang H, Park IY, Walker C, Wu P, Laezza F, Sowers LC, Zhang KL (2015). Quantification of histone modifications by parallel-reaction monitoring: a method validation. Analytical Chemical.

[ref-44] Sun Q, Li JM, Sun Y, Chen Q, Zhang L, Le T (2020). The antifungal effects of cinnamaldehyde against *Aspergillus niger* and its application in bread preservation. Food Chemistry.

[ref-45] Sun Q, Shang B, Wang L, Lu ZS, Liu Y (2016). Cinnamaldehyde inhibits fungal growth and aflatoxin B1 biosynthesis by modulating the oxidative stress response of *Aspergillus flavus*. Applied Microbiology and Biotechnology.

[ref-46] Méchin V, Damerval C, Zivy M (2007). Total protein extraction with TCA-acetone. Methods in Molecular Biology.

[ref-47] Tomassetti M, Garavaglia BS, Vranych CV, Gottig V, Ottado J, Gramajo H, Diacovich L (2018). 3-methylcrotonyl Coenzyme A (CoA) carboxylase complex is involved in the *Xanthomonas citri* subsp. citri lifestyle during citrus infection. PLOS ONE.

[ref-48] Uddin R, Siddiqui QN, Sufian M, Azam SS, Wadood A (2019). Proteome-wide subtractive approach to prioritize a hypothetical protein of XDR-*Mycobacterium tuberculosis* as potential drug target. Genes & Genomics.

[ref-49] Usta J, Kreydiyyeh S, Barnabe P, Bou-Moughlabay Y, Nakkash-Chmaisse H (2003). Comparative study on the effect of Cinnamaon and clove extracts and their main components on different types of ATPases. Human & Experimental Toxicology.

[ref-50] Wang Q, Liu M, Xu L, Wu Y, Huang Y (2018a). Transcriptome analysis reveals the molecular mechanism of hepatic fat metabolism disorder caused by Muscovy duck reovirus infection. Avian Pathology.

[ref-51] Wang Y, Feng KW, Yang HH, Zhang ZW, Yuan YH, Yue TL (2018b). Effect of Cinnamaldehyde and Citral combination on transcriptional profile, growth, oxidative damage and patulin biosynthesis of *Penicillium expansum*. Frontiers in Microbiology.

[ref-52] Xia HL, Feng LF, Lin LJ, Jiang ZQ, Chen JQ, Shi W, Ying SB, Yu M, Ju L, Zhu LJ, Shi L, Zhang X, Lou JL (2021). Exploration of identifying novel serum biomarkers for malignant mesothelioma using iTRAQ combined with 2D-LC-MS/MS. Environmental Research.

