# Supplementary material for: Cinnamaldehyde inhibits the growth of Phytophthora capsici through disturbing metabolic homoeostasis
Source: PeerJ. 2021 Apr 30;9:e11339. doi: 10.7717/peerj.11339 (PMC8092109; doi:10.7717/peerj.11339)
Supplement: Supplemental Information 1 [file peerj-09-11339-s001.docx]

| Gene name | Forward primer | Reverse primer |
| --- | --- | --- |
| CAMK/CAMK1 protein kinase | TTTGACCGGATCGTGGAGAA | GTTGGCGTCATGGCAATACT |
| Glucan 1,3-beta-glucosidase | GGCCCTTGTAGCGATTGATG | ACCACCACGTTTGAACACTG |
| 1,3-beta-glucanosyltransferase | TGCCGTACTCAAGCAGAAGA | ACTCAACCGGTCACCAAGAA |
| Cellulose synthase 3 | CTCTGTCCGTGAACTCCGTA | CCACGGTCACGGTAGTCATA |
| Acyl-CoA dehydrogenase | AACACGGCAAGAAGATCGTG | AGAGATGGTAGCGGTCATGG |
| 3-ketoacyl-CoA thiolase | CGGACTTTGCGACATGTTCT | TGGGTGGAATGCAGGAGATT |
| Acyl-CoA dehydrogenase | AAACTCTCCGATTGCTGTGC | TCACCCTGACGGATGTGAAA |
| Acetyl-CoA carboxylase | TGGTGCCATTCAGGAGCTTA | GCTCCACGAGAACAAGTGAC |
| Fatty acid synthase subunit alpha | CTGTCTACGCTACCTGCTGA | GGTCCAAGTACACGTTGCTC |
| Elongation of fatty acids protein | TACGCCACCATCGTACTCAA | ACGAACTGGATGAGCTGGAT |
| Isovaleryl-CoA dehydrogenase | CAAGCTCTTCCGTGAACTGG | ACAGCATTGAGTGCGCTAAG |
| Methylcrotonoyl-CoA carboxylase | GTGGTCGGACGATTCTTTGG | GACACTTCGGCCTTGTCATC |
| G3PDH | AAGGACGACGAAGGAAAGGT | ACGGCATGTGCCTTAACATC |
