# Supplementary material for: Cinnamaldehyde inhibits the growth of Phytophthora capsici through disturbing metabolic homoeostasis
Source: PeerJ. 2021 Apr 30;9:e11339. doi: 10.7717/peerj.11339 (PMC8092109; doi:10.7717/peerj.11339)
Supplement: Supplemental Information 2 [file peerj-09-11339-s002.docx]

| Accession | Description | Regulation level / fold change |
| --- | --- | --- |
| D0NJS3 | Diaminopimelate decarboxylase, putative | UP/1.52 |
| H3G982 | Uncharacterized protein | UP/1.33 |
| H3H379 | Ubiquitin carboxyl-terminal hydrolase | UP/1.87 |
| G4ZEU7 | Putative uncharacterized protein | UP/2.06 |
| D0NVL3 | Zinc finger CDGSH domain-containing protein 1 | UP/1.55 |
| G5ADN0 | Formate dehydrogenase | UP/1.30 |
| G4ZG68 | Putative uncharacterized protein | UP/3.52 |
| A0A060A669 | Elongation factor 1-alpha (Fragment) | UP/1.45 |
| A0A0W8D8F9 | Arachidonate 12-lipoxygenase | UP/1.37 |
| G4ZQ23 | Phosphoglycerate mutase | UP/1.30 |
| D0P0P1 | Guanylate-binding protein, putative | UP/1.38 |
| G4ZLR3 | Putative uncharacterized protein | UP/1.32 |
| H3GCK9 | Uncharacterized protein | UP/2.04 |
| H3GWU1 | Clustered mitochondria protein homolog | UP/1.38 |
| A0A0W8B8K9 | DnaJ subfamily C member 7 | UP/1.44 |
| H3GZB3 | Uncharacterized protein | UP/1.88 |
| A7XL89 | Triosephosphate isomerase/glyceraldehyde-3-phosphate dehydrogenase (Fragment) | UP/1.44 |
| G4Z9U6 | Putative uncharacterized protein | UP/2.14 |
| A0A0W8BYL4 | Uncharacterized protein | UP/1.48 |
| G4YU43 | Putative uncharacterized protein | UP/1.43 |
| V9E6L5 | Uncharacterized protein | UP/1.30 |
| G4ZPE2 | Malate dehydrogenase | UP/1.44 |
| H3GA19 | S-adenosylmethionine synthase OS=Phytophthora ramorum PE=3 SV=1 - [H3GA19_PHYRM] | UP/1.21 |
| A0A0W8CU99 | Uncharacterized protein | UP/1.55 |
| D0MWM8 | Ubiquitin-specific protease, putative | UP/1.33 |
| G4ZH77 | Malic enzyme | UP/2.21 |
| D0N0I0 | Rab5 family GTPase, putative | UP/1.29 |
| A0A0W8CAK2 | Chaperone protein DnaK | UP/1.90 |
| A0A0W8CFH6 | Glutathione peroxidase | UP/1.38 |
| V5RSG4 | Enolase (Fragment) | UP/1.57 |
| A0A0W8DW73 | Uncharacterized protein | UP/1.77 |
| A0A0W8D453 | IMP-specific 5'-nucleotidase 1 | UP/1.59 |
| H3GBJ2 | Uncharacterized protein | UP/1.43 |
| G4YQB6 | Acetolactate synthase | UP/1.51 |
| H3GHL1 | Uncharacterized protein | UP/1.32 |
| H3H4U3 | Uncharacterized protein | UP/1.25 |
| A0A0W8DF25 | Uncharacterized protein | UP/1.23 |
| H3GVX5 | Uncharacterized protein | UP/2.10 |
| H3G9K6 | Uncharacterized protein | UP/1.41 |
| D0MRJ0 | Acetyl-coenzyme A synthetase | UP/1.55 |
| W2NFW6 | Translation initiation factor aIF-2 | UP/1.38 |
| W2HBR6 | Uncharacterized protein | UP/1.42 |
| A0A0W8CUU1 | Uncharacterized protein | UP/1.90 |
| H3GAL9 | Dihydrolipoyl dehydrogenase | UP/1.26 |
| H3GHZ1 | Uncharacterized protein | UP/7.46 |
| G4ZEV9 | Putative uncharacterized protein | UP/1.94 |
| H3GS31 | Uncharacterized protein | UP/1.54 |
| H3G7T7 | Uncharacterized protein | UP/1.26 |
| D0P462 | Mannitol dehydrogenase, putative | UP/5.18 |
| W2IU60 | Cytochrome b-c1 complex subunit 6 | UP/1.52 |
| D0MU64 | Putative uncharacterized protein | UP/1.61 |
| V9E8R5 | Uncharacterized protein | UP/1.36 |
| A0A0W8CZ82 | Coiled-coil domain-containing protein 39 | UP/1.46 |
| D0MRF7 | Cytochrome oxidase assembly protein, putative | UP/1.52 |
| G4ZTS6 | Putative uncharacterized protein | UP/1.28 |
| D0MRX0 | 60S ribosomal protein L30, putative | UP/1.31 |
| D0NL09 | Cysteine synthase | UP/1.28 |
| G4ZVC7 | Putative uncharacterized protein | UP/1.34 |
| A0A080ZD09 | Uncharacterized protein | UP/3.16 |
| G4ZTV2 | Putative uncharacterized protein | UP/1.33 |
| H3G6S6 | Uncharacterized protein | UP/1.51 |
| A0A0W8D6X1 | Elongation factor 3 | UP/1.62 |
| H3GS96 | Uncharacterized protein | UP/1.35 |
| A0A0W8C3V9 | NADH dehydrogenase | UP/1.42 |
| A0A0W8D0J8 | Uncharacterized protein | UP/1.40 |
| H3G883 | Transaldolase | UP/1.36 |
| G4ZT06 | Cysteine synthase | UP/1.29 |
| H3GYR4 | Uncharacterized protein | UP/1.45 |
| G5A778 | Putative uncharacterized protein | UP/1.47 |
| H3GIY2 | Uncharacterized protein | UP/1.22 |
| D0NT29 | Putative uncharacterized protein | UP/1.25 |
| H3GNP1 | Uncharacterized protein | UP/1.50 |
| W2HD13 | Uncharacterized protein (Fragment) | UP/1.29 |
| D0NL60 | Short chain dehydrogenase, putative | UP/1.61 |
| A0A0W8DZG3 | Aspartokinase | UP/1.44 |
| D0N666 | Ribonucleoside-diphosphate reductase | UP/1.21 |
| D0NW94 | Iron-sulfur cluster assembly protein, putative | UP/1.22 |
| A0A0W8DMF1 | COBW domain-containing protein 1 | UP/1.43 |
| G5A1I8 | Putative uncharacterized protein | UP/2.33 |
| A0A0W8DEB0 | Uncharacterized protein | UP/1.65 |
| G5AID4 | Putative uncharacterized protein | UP/2.62 |
| H3GA16 | Ribonucleoside-diphosphate reductase | UP/1.45 |
| G5AB13 | Putative uncharacterized protein | UP/1.31 |
| A0A0W8C2P0 | Transportin-1 | UP/1.93 |
| H3GTQ0 | Uncharacterized protein | UP/2.28 |
| H3GP66 | tRNA-dihydrouridine synthase | UP/1.76 |
| G5AH66 | Putative uncharacterized protein | UP/1.98 |
| H3GFB3 | Uncharacterized protein | UP/1.75 |
| H3GFG1 | Uncharacterized protein | UP/1.88 |
| A0A081ALM8 | Elongation of fatty acids protein | UP/3.07 |
| E6PBR1 | Bifunctional dethiobiotin synthetase/adenosylmethionine-8-amino-7-oxononanoate aminotransferase | UP/3.00 |
| A0A080Z8F3 | Uncharacterized protein | UP/1.36 |
| H3GAS7 | Uncharacterized protein | UP/1.36 |
| G4ZQ96 | Putative uncharacterized protein | UP/1.68 |
| W2I1P3 | Iron-sulfur cluster assembly enzyme ISCU, mitochondrial | UP/1.34 |
| A0A0W8B3K2 | Zinc-binding alcohol dehydrogenase domain-containing protein 2 | UP/1.85 |
| G4ZQ66 | Serine hydroxymethyltransferase | UP/1.30 |
| G4ZGI3 | Cystathionine beta-synthase | UP/1.20 |
| W2G0C2 | Nitrite reductase [NAD(P)H], large subunit | UP/1.75 |
| G4Z031 | Putative uncharacterized protein | UP/1.95 |
| Q6WEV3 | Glyceraldehyde-3-phosphate dehydrogenase (Fragment) | UP/2.11 |
| G4YE49 | Putative uncharacterized protein | UP/1.95 |
| G4YF85 | Putative uncharacterized protein | UP/1.68 |
| D0MYZ3 | Putative uncharacterized protein | UP/1.27 |
| A0A0W8DTX0 | Beta-adrenergic receptor kinase 1 | UP/1.68 |
| D0NFB4 | Putative uncharacterized protein | UP/1.25 |
| D0P1S9 | Putative uncharacterized protein | UP/1.63 |
| G5A116 | Putative uncharacterized protein | UP/1.72 |
| W2YYK5 | Uncharacterized protein | UP/1.53 |
| V9FDK9 | Uncharacterized protein (Fragment) | UP/1.32 |
| A0A0W8C2P9 | Cytosolic Fe-S cluster assembly factor NUBP1 homolog | UP/1.84 |
| A0A0W8CTF5 | Uncharacterized protein | UP/1.27 |
| H3G7G0 | Superoxide dismutase | UP/1.51 |
| H3GSF0 | Uncharacterized protein | UP/1.58 |
| H3GWZ5 | Acyl-coenzyme A oxidase | UP/3.02 |
| G4YYD6 | Putative uncharacterized protein | UP/1.42 |
| H3GA24 | Uncharacterized protein | UP/2.29 |
| H3GTJ1 | Coatomer subunit beta | UP/1.38 |
| W2KJT7 | Uncharacterized protein | UP/1.35 |
| G4ZYT4 | Putative uncharacterized protein (Fragment) | UP/1.31 |
| V9E086 | Uncharacterized protein | UP/1.72 |
| G4ZCU5 | Putative uncharacterized protein | UP/1.61 |
| G4YQ91 | Membrane alanine aminopeptidase, Zn-binding site | UP/1.21 |
| D0NGC0 | Putative uncharacterized protein | UP/2.12 |
| G4YSP1 | Putative uncharacterized protein | UP/1.34 |
| H3GST3 | Uncharacterized protein | UP/1.53 |
| G4ZI80 | Putative uncharacterized protein | UP/1.62 |
| H3GGJ0 | Uncharacterized protein | UP/1.51 |
| H3GRV9 | Uncharacterized protein | UP/1.26 |
| G4Z8J6 | Triosephosphate isomerase | UP/1.58 |
| A0A0W8DGA2 | Uncharacterized protein | UP/1.36 |
| D0RM25 | Ribosomal protein S6 kinase alpha-5, putative | UP/1.41 |
| G4YRR8 | Putative uncharacterized protein (Fragment) | UP/1.26 |
| G5AFD5 | Putative uncharacterized protein | UP/1.34 |
| W2NMD0 | Uncharacterized protein | UP/1.40 |
| D0NME3 | Malate dehydrogenase | UP/2.59 |
| D0MYC5 | Pyruvate, phosphate dikinase | UP/1.84 |
| G5AFA6 | Putative uncharacterized protein | UP/1.53 |
| A0A0W8DQ06 | Chaperonin CPN60-1 | UP/1.38 |
| G4YSS1 | Putative uncharacterized protein | UP/1.33 |
| A0A0W8D3G0 | H/ACA ribonucleoprotein complex subunit 4 | UP/1.46 |
| H3G8G6 | Uncharacterized protein | UP/1.22 |
| G4YH85 | Pyruvate dehydrogenase E1 component subunit alpha | UP/1.43 |
| W2P583 | Uncharacterized protein | UP/1.29 |
| H3GAR1 | Uncharacterized protein | UP/1.59 |
| H3G789 | Uncharacterized protein | UP/1.35 |
| A0A0W8CCB8 | Arg11 protein | UP/1.27 |
| G4ZRQ2 | Putative uncharacterized protein | UP/1.31 |
| H3G901 | Uncharacterized protein | UP/1.93 |
| V9E9T7 | Uncharacterized protein | UP/1.38 |
| D0NVZ1 | Developmentally-regulated GTP-binding protein 1 | UP/1.38 |
| A0A0W8DCD2 | Cyclin-dependent kinase D-1 | UP/1.26 |
| D0MY04 | Alanine aminotransferase 2 | UP/1.54 |
| A0A0W8DA02 | ATP-dependent RNA helicase DDX47 | UP/1.48 |
| G4Z1P3 | Putative uncharacterized protein | UP/1.33 |
| D0NQR5 | Aminotransferase, putative | UP/1.24 |
| G4YYJ9 | Putative uncharacterized protein | UP/1.52 |
| H3GF31 | Uncharacterized protein | UP/1.47 |
| A0A0W8CTE3 | Adenylate kinase | UP/1.31 |
| D0MSH5 | 12-oxophytodienoate reductase, putative | UP/1.46 |
| G4ZCT3 | Putative uncharacterized protein | UP/1.84 |
| A0A0W8DLV7 | Uncharacterized protein | UP/1.89 |
| A0A0W8C395 | Fatty acid synthase subunit alpha | UP/2.16 |
| V9FJT7 | Uncharacterized protein | UP/2.80 |
| A0A0W8DP59 | Uncharacterized protein | UP/1.43 |
| G5AHH4 | Putative uncharacterized protein | UP/3.14 |
| W2KMC8 | Uncharacterized protein | UP/1.65 |
| W2JGE6 | Uncharacterized protein | UP/2.32 |
| A0A0W8C8H9 | Pyridoxal biosynthesis lyase PdxS | UP/2.06 |
| H3G6G4 | Uncharacterized protein | UP/1.83 |
| H3H2W8 | Uncharacterized protein | UP/3.24 |
| D0MT62 | D-isomer specific 2-hydroxyacid dehydrogenase, putative | UP/1.26 |
| H3GP49 | Uncharacterized protein | UP/1.30 |
| G4YZE2 | Putative uncharacterized protein | UP/1.25 |
| D0NPW4 | Glutamyl-tRNA synthetase, putative | UP/1.33 |
| G4Z2C5 | Putative uncharacterized protein | UP/1.83 |
| G4YWP8 | Putative uncharacterized protein | UP/1.24 |
| D0NLW7 | Cystathionine beta-lyase | UP/1.77 |
| A7XIS7 | Enolase (Fragment) | UP/1.46 |
| D0MZX1 | Putative uncharacterized protein | UP/1.20 |
| A0A0W8CLX3 | Ubiquitin carboxyl-terminal hydrolase | UP/1.56 |
| H3GXQ9 | Uncharacterized protein | UP/1.39 |
| H3HD56 | Uncharacterized protein | UP/1.36 |
| H3H6P0 | Uncharacterized protein | UP/1.79 |
| A0A0W8DWF9 | Uncharacterized protein | UP/1.34 |
| H3G862 | Uncharacterized protein | UP/1.51 |
| A0A081B4R7 | Uncharacterized protein | UP/1.22 |
| A0A0W8DEG9 | Elicitin protein RAL13D | UP/1.40 |
| H3GQW8 | Uncharacterized protein | UP/2.54 |
| G4ZEK8 | Putative uncharacterized protein (Fragment) | UP/1.25 |
| A0A0W8CW81 | Malic enzyme | UP/1.27 |
| W2G487 | Uncharacterized protein | UP/1.34 |
| G4YKA6 | Putative uncharacterized protein | UP/1.34 |
| G4Z666 | Putative uncharacterized protein | UP/2.12 |
| D0NH58 | Midasin | UP/1.47 |
| A0A0Y0BY36 | Ribosomal protein S4 | UP/1.66 |
| G4ZQN0 | Putative uncharacterized protein | UP/1.29 |
| G5A5R8 | Putative uncharacterized protein | UP/1.45 |
| A0A0W8CK20 | Peroxiredoxin-2 | UP/3.65 |
| G4YVW9 | Putative uncharacterized protein (Fragment) | UP/1.36 |
| H3GVK0 | Uncharacterized protein | UP/2.31 |
| D0MRW1 | Naringenin,2-oxoglutarate 3-dioxygenase, putative | UP/1.54 |
| A0A0W8DGP2 | Cullin-1 | UP/1.25 |
| G5A1B5 | Putative uncharacterized protein | UP/1.73 |
| D0NH87 | Alcohol dehydrogenase, putative | UP/4.24 |
| E3T2G3 | MaoC-like dehydratase | UP/1.31 |
| A0A0W8DS29 | Mitochondrial substrate carrier family protein B | UP/1.70 |
| H3GS99 | Uncharacterized protein | UP/3.23 |
| G4YKJ0 | Putative uncharacterized protein | UP/1.73 |
| G4YPV0 | Putative uncharacterized protein | UP/2.04 |
| A0A0W8CB64 | N-alpha-acetyltransferase 16 | UP/1.44 |
| H3GUJ5 | Uncharacterized protein | UP/8.68 |
| G4ZUG8 | Putative uncharacterized protein | UP/1.39 |
| H3GRZ9 | Uncharacterized protein | UP/1.46 |
| G4YL77 | Putative uncharacterized protein | UP/1.90 |
| D0NWT0 | Elongation factor Ts, mitochondrial | UP/1.21 |
| G4YJQ5 | DAHP synthetase phospho-2dehydro-3-deoxyheptonate aldolase | UP/1.21 |
| H3G8F2 | Uncharacterized protein | UP/1.34 |
| A0A081B3N6 | Uncharacterized protein (Fragment) | UP/2.08 |
| H3GW92 | Uncharacterized protein | UP/1.71 |
| W2FVX0 | DNA-directed RNA polymerase subunit beta | UP/1.37 |
| W2H113 | Formate dehydrogenase | UP/1.62 |
| A0A0W8D6I1 | Uncharacterized protein | UP/2.11 |
| A0A0W8DTY5 | Aspartate aminotransferase | UP/2.41 |
| W2MQQ6 | Uncharacterized protein | UP/1.36 |
| G4ZZ35 | Ribonucleoside-diphosphate reductase | UP/1.29 |
| G5A275 | Putative uncharacterized protein | UP/3.36 |
| A0A0W8CY50 | E3 ubiquitin-protein ligase | UP/1.25 |
| G4YPV5 | Putative uncharacterized protein | UP/2.25 |
| A0A0W8DSC5 | Histone deacetylase complex subunit | UP/1.49 |
| A0A081A3Y9 | Uncharacterized protein | UP/1.53 |
| H3H0T2 | Uncharacterized protein | UP/2.50 |
| H3GG53 | Uncharacterized protein | UP/1.64 |
| A0A0W8C5H0 | Voltage-gated potassium channel subunit beta | UP/2.14 |
| D0MZU0 | Pyruvate dehydrogenase E1 component subunit alpha | UP/1.45 |
| H3GM98 | Uncharacterized protein | UP/2.13 |
| W2MNE0 | Uncharacterized protein | UP/1.67 |
| D0N4T2 | Putative uncharacterized protein | UP/1.32 |
| G4ZCR6 | Putative uncharacterized protein | UP/1.88 |
| A0A0W8CQC9 | Uncharacterized protein | UP/1.50 |
| A0A080ZHC9 | Uncharacterized protein | UP/1.25 |
| W2ZR72 | Uncharacterized protein | UP/1.58 |
| H3GUX4 | Uncharacterized protein | UP/1.41 |
| G4ZMK2 | Putative uncharacterized protein | UP/1.36 |
| W2NBT7 | Uncharacterized protein | UP/1.71 |
| A0A0W8BW33 | Vacuolar protein sorting-associated protein 27 | UP/1.31 |
| W2I5G6 | Glutamate-tRNA ligase | UP/1.21 |
| G5A1I5 | Putative uncharacterized protein | UP/2.83 |
| D0MY66 | Inosine-5'-monophosphate dehydrogenase, putative | UP/1.33 |
| H3GZ29 | Uncharacterized protein | UP/1.26 |
| A0A0X9GQ17 | Polyunsaturated fatty acid delta-6 desaturase | UP/1.85 |
| A0A0W8CN64 | Cell 5A endo-1 | UP/1.64 |
| A7XKI7 | Enolase (Fragment) | UP/1.44 |
| A0A081AXB3 | Uncharacterized protein | UP/1.22 |
| G5AA61 | Putative uncharacterized protein | UP/1.71 |
| A0A0W8CWX8 | D-2-hydroxyglutarate dehydrogenase | UP/1.34 |
| A0A0W8C6L7 | GMP reductase | UP/1.29 |
| H3G7G2 | NADH-cytochrome b5 reductase | UP/1.55 |
| D0N7V8 | Putative uncharacterized protein | UP/3.41 |
| H3GWU6 | Uncharacterized protein | UP/1.20 |
| H3H2D3 | Uncharacterized protein | UP/1.81 |
| W2IKJ8 | Uncharacterized protein (Fragment) | UP/1.66 |
| A7XJC6 | Enolase (Fragment) | UP/1.38 |
| G5A9X7 | Putative uncharacterized protein | UP/1.44 |
| W2HNR7 | Chorismate synthase | UP/1.73 |
| W2M1Q9 | Uncharacterized protein | UP/1.71 |
| W2JAG7 | Uncharacterized protein | UP/1.79 |
| D0N1D7 | Putative uncharacterized protein | UP/1.45 |
| G4ZC23 | Eukaryotic translation initiation factor 3 subunit A | UP/1.27 |
| A0A0W8DT71 | Dihydrolipoamide acetyltransferase component of pyruvate dehydrogenase complex | UP/1.20 |
| D0NCE4 | Small ubiquitin-like modifier (SUMO), putative | UP/1.35 |
| W2L334 | Chorismate mutase | UP/1.27 |
| H3GH18 | Uncharacterized protein | UP/1.30 |
| G5A5B0 | Putative uncharacterized protein | UP/1.37 |
| V9EJC6 | Uncharacterized protein | UP/1.25 |
| D0MWF5 | T-complex protein 1 subunit theta | UP/1.29 |
| G4YNA8 | Putative uncharacterized protein | UP/1.37 |
| A0A0W8CHV3 | Peptidyl-prolyl cis-trans isomerase | UP/1.32 |
| D0NHA7 | Putative uncharacterized protein | UP/1.31 |
| A0A081ALX9 | ATP-dependent chaperone ClpB | UP/2.54 |
| V9EI61 | Uncharacterized protein | UP/1.69 |
| G4ZTA9 | Putative uncharacterized protein | UP/3.29 |
| V9EK33 | Glycerol-3-phosphate dehydrogenase [NAD(+)] | UP/2.38 |
| W2GUU5 | Uncharacterized protein | UP/1.53 |
| G5A343 | Putative uncharacterized protein | UP/1.53 |
| A0A0W8CTN1 | Uncharacterized protein | UP/1.25 |
| D0NPB6 | Riboflavin biosynthesis protein ribBA, putative | UP/1.97 |
| W2RA78 | Uncharacterized protein | UP/1.47 |
| A0A0W8DZ09 | 6-phosphofructo-2-kinase/fructose-2 | UP/2.02 |
| A0A0W8DP01 | Uncharacterized protein | UP/1.64 |
| D0NAY8 | Isoleucyl-tRNA synthetase | UP/1.78 |
| W2IDF1 | ADP/ATP translocase 1 | UP/1.33 |
| G4ZM16 | Spindle pole body component (Fragment) | UP/1.44 |
| G5ADP1 | Putative uncharacterized protein | UP/1.42 |
| G4YGB4 | Putative uncharacterized protein | UP/1.41 |
| G4ZUB2 | Putative uncharacterized protein | UP/1.59 |
| A0A0W8C0R1 | Endo-1 | UP/1.62 |
| W2JIY1 | Uncharacterized protein (Fragment) | UP/1.45 |
| H3G875 | Uncharacterized protein | UP/2.36 |
| W2ITZ9 | Uncharacterized protein | UP/1.96 |
| D0MXR3 | Exportin-2-like protein | UP/2.18 |
| A0A0W8DXM5 | FACT complex subunit spt16 | UP/1.21 |
| H3G5L7 | Adenylyl-sulfate kinase | UP/1.46 |
| H3GB43 | Uncharacterized protein | UP/1.29 |
| H3G8H8 | Adenylosuccinate lyase | UP/1.39 |
| A0A0W8C654 | FUN14 domain-containing protein 1 | UP/1.58 |
| H3G8A9 | Uncharacterized protein | UP/1.82 |
| H3GAP7 | Uncharacterized protein | UP/2.03 |
| H3HDI3 | Uncharacterized protein | UP/1.37 |
| G4ZXQ0 | Putative uncharacterized protein | UP/2.43 |
| W2ZMZ0 | Uncharacterized protein | UP/1.43 |
| D0NQ85 | Phosphate acetyltransferase | UP/3.80 |
| A0A0W8CZK1 | Uncharacterized protein | UP/2.07 |
| H3GGA9 | Uncharacterized protein | UP/1.58 |
| A0A0W8DC37 | 60S ribosomal protein L27a | UP/1.46 |
| V9DZI9 | AGC/RSK/RSKP90 protein kinase | UP/1.23 |
| D0P461 | Mannitol dehydrogenase, putative | UP/1.71 |
| H3GAQ5 | Uncharacterized protein | UP/1.35 |
| D0NLV7 | Trans-acting enoyl reductase, putative | UP/1.66 |
| H3GG74 | Glycerol-3-phosphate dehydrogenase | UP/1.34 |
| D0NHQ6 | Putative uncharacterized protein | UP/1.57 |
| H3H7L0 | Uncharacterized protein | UP/2.83 |
| Q93VK2 | Pyruvate, phosphate dikinase | UP/1.59 |
| A0A0W8C194 | Midasin | UP/1.25 |
| H3GAG1 | Eukaryotic translation initiation factor 3 subunit I | UP/1.37 |
| D0NF06 | Acyl-coenzyme A oxidase | UP/1.27 |
| V9FE70 | Acyl-coenzyme A oxidase | UP/1.38 |
| A0A0W8D0H6 | Zinc-binding alcohol dehydrogenase domain-containing protein 2 | UP/1.55 |
| G4YYL3 | Dihydrolipoamide acetyltransferase component of pyruvate dehydrogenase complex | UP/1.58 |
| W2GAZ3 | Ketol-acid reductoisomerase | UP/1.60 |
| G4Z0G9 | Putative uncharacterized protein | UP/1.28 |
| H3G9R1 | Phosphoglycerate kinase | UP/1.58 |
| W2MLQ3 | Uncharacterized protein | UP/1.32 |
| A0A0W8CK96 | Acyl-CoA synthetase family member 3 | UP/1.33 |
| A0A0W8D3R6 | Isocitrate lyase | UP/1.69 |
| W2J214 | Uncharacterized protein (Fragment) | UP/1.22 |
| A0A081A3V5 | Uncharacterized protein | UP/1.36 |
| A0A0W8C2A4 | Uncharacterized protein | UP/1.24 |
| A0A0W8DG57 | Cyclin-dependent kinase 2 | UP/1.26 |
| A0A081A343 | 3,4-dihydroxy-2-butanone-4-phosphate synthase | UP/1.52 |
| A0A0W8DFP8 | Steroid-binding protein 3 | UP/1.22 |
| D0N1B1 | Tripeptidyl-peptidase, putative | UP/1.44 |
| G4YRB3 | Putative uncharacterized protein | UP/2.47 |
| A0A0W8CCE9 | Phosphoenolpyruvate carboxykinase | UP/1.81 |
| A0A0W8CWC8 | Uncharacterized protein | UP/1.98 |
| D0N4U8 | Serine hydroxymethyltransferase | UP/1.43 |
| D0NRY9 | Peptide methionine sulfoxide reductase | UP/2.70 |
| G4YU66 | Mitochondrial substrate carrier | UP/1.47 |
| W2KBW3 | Uncharacterized protein | UP/1.26 |
| G4Z2J6 | Putative uncharacterized protein | UP/1.35 |
| G4YH70 | SURF1-like protein | UP/1.45 |
| W2M4S1 | Glycylpeptide N-tetradecanoyltransferase | UP/1.38 |
| W2P1C1 | Uncharacterized protein (Fragment) | UP/1.91 |
| H3G9S5 | Uncharacterized protein | UP/1.36 |
| H3G6W3 | Uncharacterized protein | UP/1.33 |
| H3HDJ2 | Uncharacterized protein | UP/1.21 |
| D0NX78 | Sulfite reductase [NADPH] subunit beta, putative | UP/1.52 |
| W2FLY9 | Glucokinase | UP/1.28 |
| H3GQX5 | Uncharacterized protein | UP/1.46 |
| G5AI54 | Putative uncharacterized protein | UP/1.39 |
| G4Z9P2 | Putative uncharacterized protein (Fragment) | UP/1.76 |
| H3GQ54 | Uncharacterized protein | UP/1.89 |
| A0A0W8E0U8 | Diacylglycerol O-acyltransferase 2 protein 6 | UP/2.23 |
| H3GS72 | Uncharacterized protein | UP/1.24 |
| G4Z1F7 | Putative uncharacterized protein | UP/1.34 |
| G4Z0M1 | Isocitrate dehydrogenase [NADP] | UP/1.24 |
| G4YTK3 | Putative uncharacterized protein | UP/2.14 |
| D0NP72 | Signal recognition particle subunit SRP68 | UP/1.23 |
| D0NEX4 | D-3-phosphoglycerate dehydrogenase | UP/1.27 |
| D0MS50 | Mannitol dehydrogenase, putative | UP/1.54 |
| G4YKD9 | Pyruvate, phosphate dikinase | UP/2.80 |
| G4ZTH3 | Putative uncharacterized protein | UP/1.43 |
| A0A081AST3 | Uncharacterized protein | UP/3.63 |
| A0A0W8BX74 | Asparagine synthetase | UP/1.24 |
| H3H3A1 | Uncharacterized protein | UP/1.36 |
| A0A0W8BZV2 | Uncharacterized protein | UP/1.44 |
| D0NKD1 | Putative uncharacterized protein | UP/1.39 |
| V9EXZ4 | Uncharacterized protein | UP/1.40 |
| D0MR29 | Nucleoredoxin, putative | UP/1.72 |
| W2JIG8 | Uncharacterized protein | UP/1.92 |
| A0A081A260 | Dihydrolipoyl dehydrogenase | UP/1.72 |
| A0A0W8BXB4 | LETM1 and EF-hand domain-containing protein 1 | UP/2.47 |
| H3G552 | Uncharacterized protein | UP/1.52 |
| H3G5T7 | Uncharacterized protein | UP/24.32 |
| G4ZD02 | Putative uncharacterized protein | UP/1.23 |
| G4Z9P0 | Putative uncharacterized protein | UP/1.23 |
| A0A0W8CQ99 | Exportin-2 | UP/1.57 |
| A0A081A3H5 | Serine-tRNA ligase | UP/1.39 |
| A0A081ADC6 | Uncharacterized protein | UP/1.28 |
| H3G708 | Uncharacterized protein | UP/1.26 |
| A0A0W8CT64 | Salicylate hydroxylase | UP/2.52 |
| G4ZUS4 | Deoxyhypusine hydroxylase | UP/2.17 |
| H3GYT6 | Uncharacterized protein | UP/1.42 |
| G4ZJ94 | Histidine kinase A two component receptor | UP/1.65 |
| W2QES0 | Uncharacterized protein | UP/3.59 |
| G4YIW2 | Peptidylprolyl isomerase | UP/1.28 |
| A0A0W8D2C4 | S-adenosylmethionine synthase | UP/1.48 |
| G4YWC4 | Putative uncharacterized protein | UP/1.56 |
| H3G9V7 | Branched-chain-amino-acid aminotransferase | UP/1.30 |
| D0P2R1 | Putative uncharacterized protein | UP/1.54 |
| A0A0W8C461 | Methionine aminopeptidase | UP/1.78 |
| H3H3D6 | Uncharacterized protein | UP/1.73 |
| D0N0M0 | Carbohydrate-binding protein, putative | UP/1.48 |
| E9LJ33 | Heat shock protein 90 (Fragment) | UP/1.24 |
| G4YWQ5 | Putative uncharacterized protein | UP/1.44 |
| D0NVZ6 | Putative uncharacterized protein | UP/1.35 |
| H3G902 | Uncharacterized protein | UP/1.34 |
| G5A2P0 | Putative uncharacterized protein | UP/4.27 |
| H3HCZ8 | Uncharacterized protein | UP/1.28 |
| H3GWA7 | Uncharacterized protein | UP/1.75 |
| H3GVM8 | Uncharacterized protein | UP/1.29 |
| A0A080ZH47 | Uncharacterized protein | UP/2.77 |
| A0A0W8CT89 | Uncharacterized protein | UP/1.96 |
| G4ZCL6 | Putative uncharacterized protein | UP/3.34 |
| G4ZD04 | Putative uncharacterized protein | UP/1.56 |
| A0A0W8D4E3 | BZIP transcription factor 1 | UP/1.29 |
| D0NBZ7 | Putative uncharacterized protein | UP/1.25 |
| A0A081AUA4 | Uncharacterized protein | UP/2.37 |
| G5A075 | Putative uncharacterized protein | UP/1.55 |
| H3G8L0 | Methionine aminopeptidase | UP/1.24 |
| A0A0W8DR54 | Deoxyhypusine hydroxylase | UP/1.34 |
| W2LB98 | Uncharacterized protein (Fragment) | UP/1.24 |
| D0NGA9 | Putative uncharacterized protein | UP/1.22 |
| G4YTN3 | Putative uncharacterized protein | UP/1.34 |
| W2GSD4 | Uncharacterized protein | UP/1.51 |
| D0NN32 | Putative uncharacterized protein | UP/2.47 |
| A0A188U9J1 | Heat shock protein 90 (Fragment) | UP/1.28 |
| V9FZE2 | Phosphate acetyltransferase | UP/1.29 |
| A0A080ZI27 | Uncharacterized protein | UP/1.30 |
| D0N8B4 | Phosphoglycerate mutase | UP/1.73 |
| D0P2F9 | Fructokinase, putative | UP/1.72 |
| G4ZYS4 | Putative uncharacterized protein | UP/1.37 |
| D0N4V4 | Enoyl-CoA hydratase/isomerase family, putative | UP/1.33 |
| W2LGU2 | Uncharacterized protein | UP/1.60 |
| G5ACK1 | Putative uncharacterized protein | UP/1.37 |
| G4Z7P4 | Putative uncharacterized protein OS=Phytophthora sojae (strain P6497) GN=PHYSODRAFT_494192 PE=4 SV=1 - [G4Z7P4_PHYSP] | UP/1.23 |
| A0A0W8E045 | Phosphoethanolamine N-methyltransferase | UP/1.92 |
| D0NH60 | GrpE protein homolog | UP/1.50 |
| W2GZV1 | Uncharacterized protein | UP/1.61 |
| W2NI86 | Glutamine synthetase (Fragment) | UP/1.21 |
| G4ZWT6 | Putative uncharacterized protein | UP/1.62 |
| D0N3D8 | Choline/Carnitine O-acyltransferase, putative | UP/1.23 |
| A0A080Z9A7 | Uncharacterized protein | UP/1.46 |
| D0N3E3 | Putative uncharacterized protein | UP/1.38 |
| D0NI69 | Putative uncharacterized protein | UP/1.32 |
| A0A081B0U3 | Uncharacterized protein | UP/1.62 |
| W2M2R4 | Uncharacterized protein (Fragment) | UP/2.14 |
| G4YRA3 | Putative uncharacterized protein | UP/1.42 |
| D0NCV8 | Putative uncharacterized protein | UP/1.87 |
| A0A0W8D2A6 | Uncharacterized protein | UP/1.40 |
| G5A295 | Putative uncharacterized protein | UP/2.35 |
| H3G8V7 | Methionine aminopeptidase 2 | UP/1.36 |
| G4YV61 | Nucleolar GTP-binding protein 1 | UP/1.25 |
| G5AAL8 | Putative uncharacterized protein (Fragment) | UP/1.56 |
| G4ZE42 | Putative uncharacterized protein | UP/1.82 |
| A0A0W8CQ02 | Methyltransferase protein 13 | UP/1.28 |
| A0A0W8BV48 | Eukaryotic translation initiation factor 4 gamma 2 | UP/1.27 |
| H3G9I6 | 3-isopropylmalate dehydrogenase | UP/1.30 |
| H3G6S3 | Uncharacterized protein | UP/2.33 |
| A0A0W8DP75 | BTB/POZ and MATH domain-containing protein 2 | UP/1.83 |
| G4Z3S9 | Putative uncharacterized protein | UP/1.97 |
| G4YL70 | Putative uncharacterized protein (Fragment) | UP/1.56 |
| H3GHQ2 | Uncharacterized protein | UP/1.34 |
| G4Z9U3 | Putative uncharacterized protein | UP/1.59 |
| D0MUT8 | Potassium/sodium hyperpolarization-activated cyclic nucleotide-gated channel, putative | UP/2.60 |
| G4ZXT2 | Putative uncharacterized protein | UP/1.33 |
| H3G504 | Uncharacterized protein | UP/1.52 |
| D0N6L1 | Riboflavin kinase | UP/1.39 |
| H3HE41 | Uncharacterized protein | UP/1.33 |
| H3G9V3 | Uncharacterized protein | UP/9.55 |
| T1NXG1 | Polygalacturonase | UP/1.41 |
| A0A0W8CE85 | Protein disulfide-isomerase | UP/1.23 |
| G4ZPS6 | Putative uncharacterized protein | UP/1.31 |
| D0NS95 | Ribonucleoside-diphosphate reductase small chain | UP/2.39 |
| A0A0W8C016 | Tether containing UBX domain for GLUT4 | UP/1.37 |
| G4ZXP7 | Putative uncharacterized protein | UP/1.33 |
| H3GA49 | Uncharacterized protein | UP/1.43 |
| A0A0W8DPA7 | Deoxyhypusine synthase | UP/1.69 |
| A0A080Z839 | Uncharacterized protein | UP/1.70 |
| E6PBQ9 | Bifunctional dethiobiotin synthetase/adenosylmethionine-8-amino-7-oxononanoate aminotransferase | UP/2.27 |
| G4ZPG7 | Putative uncharacterized protein | UP/1.31 |
| G5ACJ4 | Putative uncharacterized protein (Fragment) | UP/1.32 |
| G5ABR7 | Putative uncharacterized protein | UP/1.88 |
| H3H2R3 | Uncharacterized protein | UP/1.23 |
| H3H7P7 | Uncharacterized protein | UP/1.97 |
| D0N9Y4 | Glycyl-tRNA synthetase | UP/1.38 |
| A7XK68 | Triosephosphate isomerase/glyceraldehyde-3-phosphate dehydrogenase (Fragment) | UP/1.21 |
| D0N1Q2 | ATP-binding Cassette (ABC) Superfamily | UP/1.83 |
| H3G9M0 | Uncharacterized protein | UP/1.26 |
| G5A106 | Putative endo-1,3-beta-glucanase | UP/1.28 |
| D0MQN8 | Putative uncharacterized protein | UP/1.35 |
| G4Z671 | DAHP synthetase | UP/1.93 |
| H3G793 | Uncharacterized protein | UP/1.83 |
| G4YQV4 | Putative uncharacterized protein | UP/1.70 |
| A0A0W8C4Y8 | Structural maintenance of chromosomes protein | UP/1.23 |
| H3H4D2 | Uncharacterized protein | UP/1.45 |
| A0A0W8DA15 | Dihydrolipoamide acetyltransferase component of pyruvate dehydrogenase complex | UP/1.21 |
| G4ZAW1 | Putative uncharacterized protein | UP/1.24 |
| D0NH64 | Peptidylprolyl isomerase | UP/1.45 |
| W2RG23 | Uncharacterized protein | UP/1.85 |
| F6KA95 | Ribosomal protein S2 | UP/1.42 |
| A7XL16 | Enolase (Fragment) | UP/1.39 |
| G4YJ99 | Putative uncharacterized protein | UP/1.75 |
| A0A0W8DBN1 | 1-aminocyclopropane-1-carboxylate deaminase | UP/1.27 |
| A0A080ZT58 | Uncharacterized protein | UP/1.30 |
| A0A0W8BGD1 | Riboflavin synthase | UP/1.42 |
| W2J349 | Uncharacterized protein (Fragment) | UP/1.55 |
| G4Z043 | Putative uncharacterized protein | UP/2.67 |
| H3GKR7 | Uncharacterized protein | UP/1.40 |
| A0A080Z7Q8 | Uncharacterized protein | UP/1.24 |
| H3H7G5 | Uncharacterized protein | UP/1.51 |
| D0NHM8 | Chaperonin CPN60-1, mitochondrial | UP/1.34 |
| G4ZXL2 | Putative uncharacterized protein | UP/2.19 |
| H3GFF0 | Uncharacterized protein | UP/1.22 |
| W2G5U3 | Uncharacterized protein | UP/1.34 |
| A0A081AIL4 | Ribosome recycling factor | UP/1.23 |
| A0A0W8E0F6 | Uncharacterized protein | UP/1.44 |
| G5AIR8 | Putative uncharacterized protein | UP/1.39 |
| A0A0W8BV07 | Elongation factor G, mitochondrial | UP/1.67 |
| W2IDD8 | Uncharacterized protein (Fragment) | UP/4.22 |
| H3G9R7 | Uncharacterized protein | UP/1.45 |
| D0NBE5 | Putative uncharacterized protein | UP/1.29 |
| H3G5G1 | SUMO-conjugating enzyme | UP/1.33 |
| W2LE95 | Uncharacterized protein | UP/1.61 |
| D0N253 | Cytosol aminopeptidase, putative | UP/1.77 |
| W2GKT1 | 2-isopropylmalate synthase | UP/1.30 |
| D0NAT3 | Putative uncharacterized protein | UP/1.25 |
| D0NMW5 | Delta-aminolevulinic acid dehydratase | UP/1.77 |
| A0A0W8DLI1 | Ribonucleoside-diphosphate reductase small chain | UP/1.37 |
| G4YFS6 | Putative uncharacterized protein | UP/1.29 |
| A0A0W8CJE8 | Uncharacterized protein | UP/1.89 |
| G5AA60 | Putative uncharacterized protein | UP/1.23 |
| H3GWU0 | Uncharacterized protein | UP/1.43 |
| W2PTZ5 | Uncharacterized protein | UP/1.32 |
| V9EYE2 | Ribosomal protein | UP/2.01 |
| D0NXQ0 | Probable acetate kinase | UP/1.37 |
| H3GB82 | Uncharacterized protein | UP/2.71 |
| G5A9A0 | Glycerol-3-phosphate dehydrogenase [NAD(+)] | UP/2.39 |
| A0A0W8C3G4 | Formate--tetrahydrofolate ligase | UP/1.56 |
| A0A080ZLM1 | Uncharacterized protein | UP/1.35 |
| H3H6N4 | Uncharacterized protein | UP/1.47 |
| W2J1W2 | Phenylalanine-tRNA ligase | UP/1.22 |
| H3G951 | Citrate synthase | UP/1.63 |
| G4YQK7 | Putative uncharacterized protein | UP/1.27 |
| H3GGF7 | Uncharacterized protein | UP/1.29 |
| D0N3H5 | Deoxyribose-phosphate aldolase 1 | UP/1.49 |
| D0NZ18 | Acetyl-CoA carboxylase, putative | UP/1.69 |
| W2FWN7 | Uncharacterized protein | UP/1.79 |
| A0A081AJM2 | Uncharacterized protein | UP/1.26 |
| A0A0W8BZR3 | Tyrosine--tRNA ligase | UP/1.34 |
| A0A0W8CSY7 | Uncharacterized protein | UP/1.82 |
| G5A1C3 | Putative uncharacterized protein | UP/1.86 |
| G5AA29 | Putative uncharacterized protein | UP/1.23 |
| H3GD81 | Uncharacterized protein | UP/1.40 |
| H3GI11 | Uncharacterized protein | UP/1.24 |
| G5A241 | Putative uncharacterized protein | UP/2.26 |
| A0A0W8D253 | Arabinan endo-1 | UP/1.66 |
| H3G8J8 | Cysteine synthase | UP/1.55 |
| G4ZTA5 | Putative uncharacterized protein | UP/2.18 |
| A0A0W8D396 | Lipoyl synthase, mitochondrial | UP/1.44 |
| W2K7F5 | Uncharacterized protein | UP/1.44 |
| A0A081B0U5 | Uncharacterized protein | UP/1.61 |
| H3H329 | Uncharacterized protein | UP/3.45 |
| H3GMJ1 | Uncharacterized protein | UP/1.41 |
| A0A080ZZF7 | Uncharacterized protein | UP/1.34 |
| H3G900 | Uncharacterized protein | UP/1.80 |
| W2Q5G2 | CMGC/CDK/CDC2 protein kinase | UP/1.31 |
| H3H2X5 | Uncharacterized protein | UP/1.22 |
| G4ZZC9 | Putative uncharacterized protein | UP/1.42 |
| V9E9R7 | Uncharacterized protein | UP/1.52 |
| H3GC79 | Uncharacterized protein | UP/1.25 |
| A0A0W8DUL4 | Uncharacterized protein | UP/1.36 |
| G4YZZ5 | Putative uncharacterized protein | UP/1.91 |
| D0MYL3 | U3 small nucleolar RNA-associated protein, putative | UP/1.43 |
| H3G9G9 | Uncharacterized protein | UP/3.45 |
| D0N0M6 | Putative uncharacterized protein | UP/1.20 |
| A0A0W8BFZ1 | Uncharacterized protein | UP/1.74 |
| V9DUW3 | Glucokinase (Fragment) | UP/1.56 |
| W2KK30 | Uncharacterized protein (Fragment) | UP/2.92 |
| A0A0W8D3J5 | Uncharacterized protein | UP/1.38 |
| D0NLI0 | Putative uncharacterized protein | UP/1.51 |
| W2FY27 | Chaperone DnaJ | UP/1.23 |
| H3G6E8 | Uncharacterized protein | UP/1.32 |
| G4ZPB2 | UMP-CMP kinase | UP/1.50 |
| W2IVN8 | Clathrin light chain | UP/1.67 |
| Q8H706 | Transaldolase | UP/1.25 |
| W2JZK7 | Uncharacterized protein (Fragment) | UP/1.24 |
| V9FHJ4 | Uncharacterized protein | UP/2.56 |
| G4YNG4 | Putative uncharacterized protein | UP/1.21 |
| A0A0W8CFW8 | Uncharacterized protein | UP/1.27 |
| G5ABI6 | Putative uncharacterized protein | UP/1.40 |
| G4ZZL2 | Putative uncharacterized protein | UP/1.76 |
| A0A0W8C9V7 | Uncharacterized protein | UP/1.56 |
| D0NMV9 | Hybrid signal transduction histidine kinase, putative | UP/1.20 |
| D0NAR3 | 12-oxophytodienoate reductase, putative | UP/2.25 |
| D0NXV2 | Putative uncharacterized protein | UP/2.13 |
| D0MYW1 | Alcohol dehydrogenase, putative | UP/1.48 |
| D0NFV8 | Flavin-binding monooxygenase-like protein | UP/1.26 |
| H3GZ46 | Uncharacterized protein | UP/1.30 |
| H3GT64 | Uncharacterized protein | UP/1.33 |
| A7XLB6 | Enolase (Fragment) | UP/1.49 |
| H3GMU1 | Uncharacterized protein | UP/1.63 |
| H3GQS3 | Uncharacterized protein | UP/1.27 |
| D0NEF3 | 40S ribosomal protein S2 | UP/1.40 |
| D0NMA0 | Glutathione S-transferase, putative | UP/1.21 |
| H3GCJ4 | Uncharacterized protein | UP/1.35 |
| V9FU39 | Uncharacterized protein | UP/1.42 |
| G4Z409 | Putative uncharacterized protein | UP/1.84 |
| A7XL75 | Enolase (Fragment) | UP/1.29 |
| P27165 | Calmodulin | UP/1.53 |
| H3GM24 | Uncharacterized protein | UP/1.34 |
| A0A0W8C1S7 | Aspartate beta-hydroxylase domain-containing protein 2 | UP/1.43 |
| A0A0W8DDT1 | Uncharacterized protein | UP/1.21 |
| G4Z7H0 | Putative uncharacterized protein | UP/1.97 |
| A0A0W8CHY1 | Retinoblastoma-binding protein 5 | UP/1.22 |
| A0A080ZSB8 | Uncharacterized protein | UP/1.38 |
| D0NQ89 | Catalase | UP/2.39 |
| V9G2Y2 | Uncharacterized protein | UP/1.56 |
| G4Z2L1 | Citrate synthase | UP/1.91 |
| D0NYA4 | 3-isopropylmalate dehydratase large subunit, putative | UP/2.11 |
| D0QU52 | Cytochrome b (Fragment) | UP/1.34 |
| D0NHF9 | Putative uncharacterized protein | UP/1.35 |
| A0A0W8DZS6 | 2-hydroxyacid dehydrogenase | UP/1.29 |
| D0MZZ8 | Putative uncharacterized protein | UP/1.29 |
| A0A0U1U0C9 | NADH-ubiquinone oxidoreductase chain 1 (Fragment) | UP/1.44 |
| G4YGC0 | Putative uncharacterized protein | UP/1.50 |
| H3G6D8 | Uncharacterized protein | UP/1.35 |
| D0NY71 | Putative uncharacterized protein | UP/1.72 |
| H3GAP5 | Uncharacterized protein | UP/1.63 |
| G4ZTQ8 | Putative uncharacterized protein | UP/1.50 |
| D0NF22 | Atlastin-like protein | UP/1.46 |
| D0NHJ5 | Mitochondrial 39-S ribosomal protein L47, putative | UP/1.23 |
| A0A0W8D401 | Vacuolar membrane-associated protein iml1 | UP/1.21 |
| D0N687 | Glucokinase, putative | UP/1.73 |
| G4YTU9 | Putative uncharacterized protein | UP/1.36 |
| G4Z0N4 | NADH-cytochrome b5 reductase | UP/1.51 |
| H3H6Z4 | Uncharacterized protein | UP/1.20 |
| A0A0W8CNY0 | NEDD8-conjugating enzyme Ubc12 | UP/1.32 |
| W2X5K4 | Uncharacterized protein | UP/2.43 |
| G4ZXL0 | Putative uncharacterized protein | UP/1.26 |
| G4YTK1 | Putative uncharacterized protein | UP/1.20 |
| A0A0W8D152 | Fumarylacetoacetate hydrolase domain-containing protein 2 | UP/1.49 |
| G4Z2A3 | Putative uncharacterized protein | UP/1.25 |
| G4ZQP7 | Putative uncharacterized protein | UP/1.25 |
| D0N0B5 | COP9 signalosome complex subunit, putative | UP/1.28 |
| W2X1E3 | Uncharacterized protein (Fragment) | UP/1.35 |
| G4ZJ38 | Tyrosine biosynthesis bifunctional enzyme | UP/1.40 |
| G4Z039 | Putative uncharacterized protein | Down/0.24 |
| A0A0W8CPW0 | Uncharacterized protein | Down/0.45 |
| W2PW29 | Uncharacterized protein | Down/0.14 |
| H3G7I2 | Uncharacterized protein | Down/0.55 |
| W2X1F3 | Uncharacterized protein (Fragment) | Down/0.37 |
| A0A0W8DII0 | Uncharacterized protein | Down/0.17 |
| H3GMT5 | Uncharacterized protein | Down/0.22 |
| D0NIE3 | 4-hydroxyphenylpyruvate dioxygenase, putative | Down/0.40 |
| G5A8W9 | Autophagy protein 5 | Down/0.79 |
| G5A0U5 | Putative uncharacterized protein | Down/0.26 |
| D0NNP3 | 1,3-beta-glucanosyltransferase | Down/0.44 |
| G4ZQT1 | Putative uncharacterized protein | Down/0.27 |
| G5A452 | Putative uncharacterized protein | Down/0.42 |
| H3GL28 | Uncharacterized protein | Down/0.36 |
| D0MRI9 | Acetyl-coenzyme A synthetase | Down/0.53 |
| A0A0W8CYP2 | Alkylated DNA repair protein alkB 8 | Down/0.82 |
| G4YQZ3 | Putative uncharacterized protein | Down/0.58 |
| G4ZIY9 | Putative uncharacterized protein | Down/0.75 |
| A0A0W8DU38 | Uncharacterized protein | Down/0.43 |
| D0NSS4 | Sodium/potassium-transporting ATPase subunit alpha, putative | Down/0.18 |
| G4YY37 | Putative uncharacterized protein | Down/0.35 |
| A0A0W8D414 | Glyceraldehyde-3-phosphate dehydrogenase | Down/0.31 |
| W2HA56 | Uncharacterized protein | Down/0.49 |
| A0A080Z0A1 | Uncharacterized protein | Down/0.55 |
| D0NYU5 | Putative uncharacterized protein | Down/0.34 |
| H3GAK5 | Uncharacterized protein | Down/0.47 |
| G5A0F4 | Putative uncharacterized protein | Down/0.20 |
| G4ZZH9 | Putative uncharacterized protein | Down/0.38 |
| V9FRI5 | Uncharacterized protein | Down/0.48 |
| H3G6L3 | 4-hydroxyphenylpyruvate dioxygenase | Down/0.42 |
| H3GQP7 | Uncharacterized protein | Down/0.35 |
| A0A0W8CAA5 | Uricase | Down/0.56 |
| G4YJ18 | Peptidyl-prolyl cis-trans isomerase | Down/0.67 |
| W2LHX2 | Uncharacterized protein (Fragment) | Down/0.47 |
| D0ND75 | Putative uncharacterized protein | Down/0.26 |
| H3GBP6 | Uncharacterized protein | Down/0.50 |
| H3H441 | Uncharacterized protein | Down/0.12 |
| W2JLT4 | Uncharacterized protein | Down/0.65 |
| H3H4L2 | Uncharacterized protein | Down/0.64 |
| D0NZT9 | Serine/threonine-protein phosphatase | Down/0.57 |
| G5A1M1 | 6,7-dimethyl-8-ribityllumazine synthase | Down/0.61 |
| D0N8A1 | Isoamyl acetate-hydrolyzing esterase 1 | Down/0.62 |
| D0N3D4 | Putative GPI-anchored serine rich tenascin-like glycoprotein | Down/0.48 |
| G4YVQ4 | Putative uncharacterized protein | Down/0.27 |
| W2QN48 | Uncharacterized protein | Down/0.45 |
| A0A0A7DNE7 | Elicitin 11949 | Down/0.24 |
| W2N7R0 | Uncharacterized protein (Fragment) | Down/0.46 |
| A0A0W8DSM9 | Calcium-transporting ATPase | Down/0.32 |
| G4ZJD1 | ABC transporter ABCA1 lipid exporter family | Down/0.34 |
| D0N565 | Putative uncharacterized protein | Down/0.72 |
| H3GKD3 | Uncharacterized protein | Down/0.26 |
| G4YRZ0 | Putative uncharacterized protein | Down/0.61 |
| D0NNA7 | Mitogen-activated protein kinase | Down/0.65 |
| D0NE48 | Alpha-mannosidase | Down/0.27 |
| A0A0W8CXK6 | Uncharacterized protein | Down/0.62 |
| H3GRZ0 | Uncharacterized protein | Down/0.35 |
| D0NBH1 | Aquaporin, putative | Down/0.25 |
| A0A0W8DEH4 | Actin-related protein 2/3 complex subunit 3 | Down/0.40 |
| G4ZCH5 | Putative uncharacterized protein | Down/0.25 |
| H3G972 | Uncharacterized protein | Down/0.68 |
| G4ZAM2 | Putative uncharacterized protein (Fragment) | Down/0.75 |
| G4ZCK3 | Putative uncharacterized protein (Fragment) | Down/0.49 |
| A0A0W8DTS5 | Ribonuclease Z | Down/0.34 |
| A0A0W8D202 | C-factor | Down/0.51 |
| V9EZM8 | Uncharacterized protein | Down/0.57 |
| H3GA86 | Uncharacterized protein | Down/0.44 |
| Q52R83 | Thrombospondin type 1 repeat containing protein | Down/0.34 |
| G4YW20 | Aldehyde dehydrogenase | Down/0.74 |
| A0A081ACW6 | Uncharacterized protein | Down/0.73 |
| H3H0V2 | Uncharacterized protein | Down/0.42 |
| A0A0W8DD18 | Cytochrome P450 86A2 | Down/0.68 |
| H3GTG4 | Uncharacterized protein | Down/0.65 |
| W2GM67 | Uncharacterized protein | Down/0.79 |
| H3H1P2 | Uncharacterized protein | Down/0.59 |
| A0A0W8D8Q6 | Uncharacterized protein | Down/0.51 |
| W2NUF9 | CAMK/CAMK1 protein kinase | Down/0.53 |
| H3GDQ6 | Uncharacterized protein | Down/0.43 |
| H3G8V0 | Nucleoside diphosphate kinase | Down/0.67 |
| D0NBK9 | Putative uncharacterized protein | Down/0.33 |
| D0MUX1 | Trifunctional enzyme subunit beta, mitochondrial | Down/0.46 |
| W3A0H9 | Uncharacterized protein | Down/0.33 |
| H3GDZ7 | Uncharacterized protein | Down/0.63 |
| B0B0X8 | Actin | Down/0.51 |
| A0A0W8DDB5 | Uncharacterized protein | Down/0.56 |
| Q84LB1 | Putative uncharacterized protein | Down/0.20 |
| W2GLV8 | Uncharacterized protein (Fragment) | Down/0.45 |
| A0A0W8DY89 | Importin protein | Down/0.71 |
| A0A0W8D2X1 | Jerky protein | Down/0.50 |
| V9M526 | OCM1 (Fragment) | Down/0.28 |
| D0P3U4 | Major Facilitator Superfamily MFS | Down/0.45 |
| A0A0W8CCU0 | V-type proton ATPase subunit a | Down/0.68 |
| A0A0W8BYK1 | VIP36 protein | Down/0.59 |
| D0NI38 | Hypothetical cleavage-induced protein | Down/0.53 |
| W2KFP3 | Uncharacterized protein | Down/0.66 |
| A0A0W8BYF5 | Sulfatase protein | Down/0.62 |
| H3G860 | Uncharacterized protein | Down/0.26 |
| A0A075DCR9 | Heat shock protein 90 (Fragment) | Down/0.13 |
| W2LI88 | Uncharacterized protein | Down/0.50 |
| D0N745 | Major vault protein | Down/0.53 |
| G4YJ89 | Kinesin-like protein | Down/0.78 |
| D0NY52 | Alkaline phosphatase | Down/0.38 |
| W2XYX1 | Uncharacterized protein | Down/0.27 |
| G4Z571 | Putative uncharacterized protein | Down/0.72 |
| W2LQK1 | Uncharacterized protein | Down/0.70 |
| D0NUQ0 | Metalloprotease family M16A, putative | Down/0.49 |
| H3HDF1 | Uncharacterized protein | Down/0.65 |
| H3GZ00 | Uncharacterized protein | Down/0.35 |
| H3HE22 | Uncharacterized protein | Down/0.71 |
| A0A0W8BX09 | Uncharacterized protein | Down/0.21 |
| V9DZ54 | Uncharacterized protein | Down/0.51 |
| A0A0W8CBH6 | Uncharacterized protein | Down/0.59 |
| A0A0W8C648 | Folate-Biopterin Transporter (FBT) family | Down/0.30 |
| W2IP98 | Uncharacterized protein | Down/0.67 |
| D0MT89 | Putative uncharacterized protein | Down/0.38 |
| G4Z3A5 | Putative uncharacterized protein | Down/0.58 |
| D0NE44 | Alpha-N-acetylglucosaminidase (NAGLU), putative | Down/0.61 |
| D0N9B2 | Putative uncharacterized protein | Down/0.75 |
| A0A0W8DD57 | H-or Na-translocating F-type | Down/0.74 |
| W2NIA1 | Uncharacterized protein | Down/0.59 |
| H3GA21 | Uncharacterized protein | Down/0.50 |
| G4ZXR0 | Putative uncharacterized protein | Down/0.58 |
| W2LA64 | V-type proton ATPase proteolipid subunit (Fragment) | Down/0.56 |
| H3G8V3 | Uncharacterized protein | Down/0.82 |
| G4YWA6 | 40S ribosomal protein S3a | Down/0.61 |
| D0MY06 | Putative uncharacterized protein | Down/0.54 |
| G4ZN49 | Putative uncharacterized protein | Down/0.26 |
| W2ISB0 | Uncharacterized protein | Down/0.64 |
| W2Z3G7 | Alkaline phosphatase (Fragment) | Down/0.32 |
| G4YSW0 | Putative uncharacterized protein | Down/0.76 |
| A0A0W8DPK3 | E3 ubiquitin-protein ligase | Down/0.31 |
| H3GK68 | Uncharacterized protein | Down/0.30 |
| G4YS18 | Putative uncharacterized protein | Down/0.60 |
| H3GTJ6 | Uncharacterized protein | Down/0.42 |
| G4YZV6 | Putative uncharacterized protein | Down/0.44 |
| G4YFN1 | Putative arginine N-methyltransferase | Down/0.42 |
| D0NDF8 | Putative uncharacterized protein | Down/0.41 |
| A0A081AGM1 | Uncharacterized protein | Down/0.71 |
| H3GKL2 | Uncharacterized protein | Down/0.32 |
| H3GBP3 | Uncharacterized protein | Down/0.78 |
| G4YIF9 | Papain-like cysteine protease C1 | Down/0.50 |
| D0P357 | D-cysteine desulfhydrase, putative | Down/0.66 |
| A0A081AYL5 | Uncharacterized protein (Fragment) | Down/0.63 |
| H3GC20 | Uncharacterized protein | Down/0.69 |
| D0NTA5 | Carbohydrate-binding protein, putative | Down/0.47 |
| H3H272 | Uncharacterized protein | Down/0.64 |
| W2QK68 | Uncharacterized protein | Down/0.58 |
| W2W375 | Uncharacterized protein (Fragment) | Down/0.59 |
| D0NCC7 | Putative uncharacterized protein | Down/0.39 |
| D0NCX0 | Putative uncharacterized protein | Down/0.78 |
| D0MTR2 | AP-2 complex subunit sigma | Down/0.66 |
| D0NDD7 | Propionyl coenzyme A carboxylase (Pi-PCC1) | Down/0.55 |
| W2GQV5 | Uncharacterized protein | Down/0.57 |
| A0A081A0G2 | Cysteine-tRNA ligase | Down/0.54 |
| D0NJ83 | Putative uncharacterized protein | Down/0.63 |
| A4ZHD7 | ATP synthase subunit alpha | Down/0.55 |
| A0A0W8CPH6 | Gem-associated protein 2 | Down/0.50 |
| A0A0W8DSZ5 | Uncharacterized protein | Down/0.35 |
| G4YRP1 | Putative uncharacterized protein | Down/0.77 |
| G4YPJ1 | Putative uncharacterized protein | Down/0.50 |
| A0A0W8CQ68 | L-ectoine synthase | Down/0.51 |
| H3GAE7 | Uncharacterized protein | Down/0.68 |
| A0A0W8D739 | Uncharacterized protein | Down/0.63 |
| W2J1C1 | Uncharacterized protein (Fragment) | Down/0.60 |
| G4ZJ06 | Putative uncharacterized protein | Down/0.33 |
| D0N7Z2 | Acyl-CoA dehydrogenase, putative | Down/0.62 |
| H3GV00 | Uricase | Down/0.53 |
| W2H254 | Uncharacterized protein | Down/0.60 |
| D0MWH9 | Carbohydrate esterase, putative | Down/0.38 |
| Q0PI79 | Elicitin-like protein 5 (Fragment) | Down/0.64 |
| W2LE99 | Glycerol kinase | Down/0.58 |
| D0NEB9 | Alanyl-tRNA synthetase | Down/0.63 |
| H3GJX0 | Uncharacterized protein | Down/0.45 |
| D0NFM9 | Succinyl-CoA:3-ketoacid-coenzyme A transferase subunit A | Down/0.56 |
| D0NG88 | Putative uncharacterized protein | Down/0.62 |
| G5AB88 | Putative uncharacterized protein | Down/0.50 |
| H3GQ05 | Uncharacterized protein | Down/0.74 |
| D0N3A1 | Putative uncharacterized protein | Down/0.70 |
| D0NFC1 | ATP-binding Cassette (ABC) Superfamily | Down/0.42 |
| H3GX39 | Uncharacterized protein | Down/0.65 |
| A0A0W8D9P9 | Uncharacterized protein | Down/0.47 |
| H3GWR7 | Uncharacterized protein | Down/0.55 |
| A0A0W8D6C0 | Uncharacterized protein | Down/0.40 |
| H3GAH3 | Uncharacterized protein | Down/0.72 |
| A0A0W8CDP7 | Uncharacterized protein | Down/0.50 |
| Q697V4 | Translation elongation factor 1 alpha (Fragment) | Down/0.54 |
| H3G7C9 | Uncharacterized protein | Down/0.62 |
| D0NTI5 | Cysteine protease family C01A, putative | Down/0.55 |
| G5AIC5 | Putative uncharacterized protein | Down/0.51 |
| A0A0W8DAA0 | GrpE protein | Down/0.56 |
| A0A081A1K2 | Uncharacterized protein | Down/0.56 |
| Q5S7T1 | Putative nuclear LIM interactor-interacting protein | Down/0.52 |
| H3GFJ1 | Uncharacterized protein | Down/0.77 |
| D0NEZ5 | Alcohol dehydrogenase, putative | Down/0.27 |
| W2KKM9 | Uncharacterized protein | Down/0.63 |
| G4YQN0 | Putative uncharacterized protein | Down/0.54 |
| D0MZA4 | Putative uncharacterized protein | Down/0.35 |
| A0A0W8C4Q7 | Voltage-dependent anion-selective channel protein 3 | Down/0.64 |
| W2GA17 | Uncharacterized protein | Down/0.49 |
| G4YVL8 | Putative uncharacterized protein | Down/0.68 |
| A0A0W8CRD8 | Phosphatidylinositol kinase (PIK-L2) | Down/0.62 |
| A0A0W8BVQ3 | Alpha-mannosidase | Down/0.60 |
| G5AA22 | Family 30 glycoside hydrolase | Down/0.54 |
| W2FQZ4 | Uncharacterized protein | Down/0.71 |
| A0A0W8DSV8 | ATP-dependent RNA helicase DED1 | Down/0.67 |
| G4ZCB9 | Putative uncharacterized protein | Down/0.60 |
| G4YVV4 | Phosphoribosyldiphosphate synthetase | Down/0.79 |
| D0NCV1 | Glucan 1,3-beta-glucosidase, putative | Down/0.48 |
| A0A080Z9G4 | Diphthamide biosynthesis protein 2 | Down/0.63 |
| H3HA73 | Uncharacterized protein | Down/0.71 |
| A0A0W8DAG3 | Secreted protein | Down/0.75 |
| B5M4T9 | G protein beta subunit 1 | Down/0.58 |
| A0A0W8C456 | Uncharacterized protein | Down/0.38 |
| H3G770 | Glutaredoxin | Down/0.61 |
| H3GT38 | Uncharacterized protein | Down/0.58 |
| D0NW28 | Glycerol kinase 1 | Down/0.57 |
| A0A0W8D9Y4 | Uncharacterized protein | Down/0.80 |
| H3GCZ5 | Ammonium transporter | Down/0.46 |
| H3HB29 | Uncharacterized protein | Down/0.62 |
| H3H4I4 | Uncharacterized protein | Down/0.57 |
| H3GMD8 | Uncharacterized protein | Down/0.67 |
| G4ZVU8 | Putative uncharacterized protein | Down/0.64 |
| H3HDE8 | Uncharacterized protein | Down/0.68 |
| A0A0W8CIA6 | Zonadhesin | Down/0.63 |
| D0MZW8 | Peptidase, putative | Down/0.57 |
| G4YI13 | ABCA1 lipid exporter | Down/0.64 |
| A0A081A728 | Uncharacterized protein | Down/0.58 |
| W2JL79 | Uncharacterized protein (Fragment) | Down/0.64 |
| G4ZVG5 | Putative uncharacterized protein | Down/0.69 |
| G4YQJ9 | Putative uncharacterized protein | Down/0.47 |
| G5AFA7 | DNA ligase | Down/0.59 |
| G5A6V7 | Putative uncharacterized protein | Down/0.73 |
| W2GVB3 | Uncharacterized protein | Down/0.55 |
| W2HC17 | Uncharacterized protein | Down/0.74 |
| V9E5C2 | Uncharacterized protein | Down/0.69 |
| I1TGL3 | Tubulin beta chain (Fragment) | Down/0.76 |
| H3G7L9 | Uncharacterized protein | Down/0.54 |
| G4Z7W4 | Putative uncharacterized protein | Down/0.73 |
| A0A0W8DSV4 | ADP-ribosylation factor family | Down/0.67 |
| V9EV57 | Proteasome endopeptidase complex | Down/0.83 |
| A0A0W8DC69 | Alpha-N-acetylglucosaminidase | Down/0.58 |
| G4Z3P3 | Putative uncharacterized protein | Down/0.83 |
| H3G6X9 | Uncharacterized protein | Down/0.60 |
| D0NE99 | Putative uncharacterized protein | Down/0.71 |
| W2KQJ6 | Uncharacterized protein (Fragment) | Down/0.69 |
| D0N7D7 | Catalase-peroxidase, putative | Down/0.34 |
| D0N1I3 | Putative uncharacterized protein | Down/0.61 |
| D0P053 | Putative uncharacterized protein | Down/0.45 |
| G4YU69 | Putative uncharacterized protein | Down/0.77 |
| D0N324 | Carbon-nitrogen hydrolase, putative | Down/0.65 |
| G4Z106 | Putative uncharacterized protein | Down/0.65 |
| H3GJ37 | Uncharacterized protein | Down/0.73 |
| G4YEW8 | Glycerol-3-phosphate dehydrogenase [NAD(+)] | Down/0.72 |
| G5AAH9 | Xylose isomerase | Down/0.50 |
| A0A0W8C0R5 | Amino Acid/Auxin Permease (AAAP) Family | Down/0.52 |
| D0MT49 | Glycoside hydrolase, putative | Down/0.54 |
| A0A0W8DAF3 | Uncharacterized protein | Down/0.75 |
| H3GB49 | Uncharacterized protein | Down/0.33 |
| A0A0W8DRB0 | Uncharacterized protein | Down/0.74 |
| V9EH02 | AGC protein kinase, variant | Down/0.56 |
| A0A0W8DSE3 | 60S acidic ribosomal protein P0 | Down/0.38 |
| A0A0W8CH75 | Uncharacterized protein | Down/0.63 |
| W2KCL1 | Uncharacterized protein | Down/0.69 |
| A0A0W8DVQ2 | Ubiquitin modifier-activating enzyme 1 | Down/0.79 |
| H3GWC9 | Uncharacterized protein | Down/0.38 |
| H3GDL0 | Uncharacterized protein | Down/0.45 |
| D0N747 | Superoxide dismutase | Down/0.39 |
| A0A0W8DK62 | Replication factor C subunit 3 | Down/0.82 |
| D0N728 | Palmitoyl-protein thioesterase 1, putative | Down/0.68 |
| H6U2P7 | Cellulose synthase 3 | Down/0.70 |
| W2QSM7 | Uncharacterized protein | Down/0.52 |
| D0NMC6 | Putative uncharacterized protein | Down/0.75 |
| A0A0W8DFS9 | Elongation factor 1-gamma 2 | Down/0.67 |
| A0A0W8D4W4 | Calcium-transporting ATPase | Down/0.60 |
| G4YUH8 | Putative uncharacterized protein | Down/0.38 |
| G4ZNF4 | Putative uncharacterized protein | Down/0.78 |
| H3GA10 | Uncharacterized protein | Down/0.56 |
| A0A0W8DP50 | Fatty acyl-CoA reductase | Down/0.67 |
| G4YQ99 | Putative uncharacterized protein | Down/0.68 |
| G4Z5K4 | Alpha-mannosidase | Down/0.57 |
| D0N5G9 | Putative uncharacterized protein | Down/0.71 |
| W2G6A2 | Uncharacterized protein | Down/0.66 |
| H3GT53 | Uncharacterized protein | Down/0.62 |
| G5A7L1 | Putative uncharacterized protein | Down/0.56 |
| W2HTQ7 | Uncharacterized protein | Down/0.55 |
| W2LJG5 | Uncharacterized protein | Down/0.67 |
| H3GYQ0 | Uncharacterized protein | Down/0.63 |
| G5AET7 | Putative uncharacterized protein (Fragment) | Down/0.59 |
| W2QV90 | Uncharacterized protein | Down/0.72 |
| W2HC46 | Urease | Down/0.70 |
| A0A0W8D4H7 | MPN domain-containing protein | Down/0.74 |
| W2H9P7 | Uncharacterized protein | Down/0.80 |
| G4ZCI9 | Putative uncharacterized protein | Down/0.75 |
| G4YQC5 | Putative uncharacterized protein | Down/0.75 |
| D0MUR7 | Putative chitin synthase (Chitin-UDP-GlcNac-transferase) | Down/0.64 |
| V9EB37 | Uncharacterized protein | Down/0.45 |
| H3G8D0 | Glucose-6-phosphate isomerase | Down/0.64 |
| D0NPV1 | Cytoskeleton-associated protein, putative | Down/0.65 |
| G4YL75 | Putative uncharacterized protein | Down/0.64 |
| G4YVZ2 | Putative uncharacterized protein | Down/0.81 |
| H3GQU5 | Uncharacterized protein | Down/0.73 |
| G4YH17 | Putative uncharacterized protein | Down/0.64 |
| G4ZAQ5 | Putative uncharacterized protein | Down/0.81 |
| A0A0W8DAU2 | Serine/threonine-protein kinase drkB | Down/0.74 |
| D0MXZ3 | Serine protease family S01B, putative | Down/0.78 |
| A0A081A8P0 | Phosphoribosylformylglycinamidine synthase | Down/0.76 |
| H3GFD7 | Uncharacterized protein | Down/0.63 |
| W2HU14 | Proteasome subunit beta type | Down/0.82 |
| G4ZS20 | Putative uncharacterized protein | Down/0.66 |
| G4Z2Q7 | Putative uncharacterized protein | Down/0.56 |
| A0A0W8DAV8 | Uncharacterized protein | Down/0.57 |
| G5A5E0 | Putative uncharacterized protein | Down/0.69 |
| D0N088 | Regulator of chromosome condensation (RCC1)-like protein | Down/0.59 |
| H3G780 | Uncharacterized protein | Down/0.64 |
| A0A080ZDB9 | Carboxypeptidase | Down/0.61 |
| H3H1Q7 | Uncharacterized protein | Down/0.57 |
| H3G9K3 | Uncharacterized protein | Down/0.73 |
| H3GU31 | Uncharacterized protein | Down/0.83 |
| A0A0W8D3F9 | Creatine kinase | Down/0.54 |
| G4YKV7 | Putative uncharacterized protein | Down/0.49 |
| A0A0W8BYL6 | Propionyl-CoA carboxylase beta chain | Down/0.74 |
| G4YU48 | Putative uncharacterized protein | Down/0.63 |
| H3H2E7 | Uncharacterized protein | Down/0.45 |
| A0A081B1X1 | Uncharacterized protein | Down/0.82 |
| D0NIC0 | Putative uncharacterized protein | Down/0.51 |
| G4YUK5 | Putative uncharacterized protein | Down/0.52 |
| A0A0W8CXC4 | WRKY transcription factor 19 | Down/0.67 |
| D0P1K9 | Glycoside hydrolase, putative | Down/0.65 |
| A0A0W8DL71 | Heat shock protein 75 kDa | Down/0.83 |
| W2G3U8 | Uncharacterized protein | Down/0.83 |
| W2K9B1 | Uncharacterized protein | Down/0.68 |
| W2J343 | Uncharacterized protein (Fragment) | Down/0.67 |
| W2KEE3 | Uncharacterized protein (Fragment) | Down/0.62 |
| W2GR41 | Uncharacterized protein | Down/0.55 |
| G5A2T3 | Putative uncharacterized protein | Down/0.69 |
| H3H3A7 | Uncharacterized protein | Down/0.76 |
| H3GPM4 | Uncharacterized protein | Down/0.40 |
| D0NVM5 | Carbohydrate esterase, putative | Down/0.50 |
| G4ZJ44 | Eukaryotic translation initiation factor 3 subunit G | Down/0.49 |
| A0A0W8CDJ6 | Uncharacterized protein | Down/0.57 |
| G5A1T0 | Putative uncharacterized protein | Down/0.32 |
| G5A9Y1 | Putative uncharacterized protein | Down/0.74 |
| G4YUB2 | Calcium-transporting ATPase | Down/0.70 |
| A0A0W8D475 | Plasma membrane ATPase | Down/0.58 |
| A0A080Z6T8 | Uncharacterized protein | Down/0.68 |
| D0NXZ9 | Secreted RxLR effector peptide protein, putative | Down/0.50 |
| W2HE20 | Uncharacterized protein | Down/0.44 |
| A0A0W8DHV5 | Histone-binding protein | Down/0.48 |
| D0P2P4 | tRNA-specific adenosine deaminase, putative | Down/0.61 |
| H3HD79 | Uncharacterized protein | Down/0.68 |
| D0NFR4 | Apolipoprotein A-I-binding protein, putative | Down/0.78 |
| G4YWJ3 | Putative uncharacterized protein | Down/0.70 |
| G4Z0E8 | Putative uncharacterized protein | Down/0.70 |
| A0A0W8DSN9 | Uncharacterized protein | Down/0.68 |
| A0A0W8DKF8 | Eukaryotic initiation factor 4A-III | Down/0.68 |
| W2MYF9 | Uncharacterized protein | Down/0.78 |
| D0N969 | Methylcrotonoyl-CoA carboxylase beta chain, mitochondrial | Down/0.69 |
| A0A080ZBH7 | Uncharacterized protein | Down/0.53 |
| G4ZG43 | Putative uncharacterized protein (Fragment) | Down/0.77 |
| W2MCK3 | Uncharacterized protein | Down/0.62 |
| H3GVZ2 | Uncharacterized protein | Down/0.53 |
| D0N114 | AP-2 complex subunit alpha, putative | Down/0.49 |
| V9EMF7 | Uncharacterized protein | Down/0.67 |
| G4YFL5 | Putative uncharacterized protein | Down/0.50 |
| A0A081AUS4 | Uncharacterized protein | Down/0.70 |
| G4YZ19 | Putative uncharacterized protein | Down/0.58 |
| H3GA63 | Xylose isomerase | Down/0.23 |
| A0A0W8CZV7 | Uncharacterized protein O | Down/0.59 |
| D0NGD1 | Putative uncharacterized protein | Down/0.82 |
| W2HV65 | Uncharacterized protein | Down/0.68 |
| H3GIP9 | Uncharacterized protein | Down/0.75 |
| D0MT54 | Argonaute3 (AGO3) | Down/0.78 |
| A0A081A7X8 | Uncharacterized protein | Down/0.74 |
| D0NK27 | Putative uncharacterized protein | Down/0.67 |
| A0A080ZSN7 | Translation elongation factor Tu, variant 3 | Down/0.69 |
| A0A0W8CS50 | Uncharacterized protein | Down/0.68 |
| G4YMH3 | Putative uncharacterized protein | Down/0.55 |
| A7XJD8 | Enolase (Fragment) | Down/0.42 |
| G4Z8W4 | Putative uncharacterized protein | Down/0.67 |
| V9E4L2 | Pentafunctional AROM polypeptide | Down/0.82 |
| A0A0W8DRF1 | Structural maintenance of chromosomes protein | Down/0.64 |
| D0NYS3 | Putative uncharacterized protein | Down/0.64 |
| D0N8F9 | Phospholipid-transporting ATPase | Down/0.59 |
| A0A0W8DQQ1 | Purple acid phosphatase | Down/0.67 |
| A0A081ASX2 | Uncharacterized protein | Down/0.59 |
| G4YRN9 | Putative uncharacterized protein | Down/0.77 |
| H3GKZ2 | Uncharacterized protein | Down/0.39 |
| W2GUP9 | TKL protein kinase | Down/0.74 |
| W2HF34 | Non-specific serine/threonine protein kinase | Down/0.68 |
| G4YXE4 | Putative uncharacterized protein | Down/0.51 |
| A0A0W8D719 | Uncharacterized protein | Down/0.72 |
| G4YJ29 | Putative uncharacterized protein | Down/0.46 |
| G4YYT8 | Putative uncharacterized protein | Down/0.35 |
| G5AEA3 | Putative uncharacterized protein | Down/0.61 |
| H3H4A6 | Uncharacterized protein | Down/0.55 |
| D0P0K7 | TRAF3-interacting protein, putative | Down/0.72 |
| D0P1T4 | Putative uncharacterized protein | Down/0.63 |
| W2NLB0 | Uncharacterized protein | Down/0.63 |
| D0NI70 | Proteasome subunit beta type | Down/0.69 |
| G4ZRL7 | Putative uncharacterized protein | Down/0.70 |
| G4ZXM3 | Putative uncharacterized protein | Down/0.49 |
| H3G5E2 | Uncharacterized protein | Down/0.79 |
| H3G966 | Uncharacterized protein | Down/0.56 |
| G4YY18 | Putative uncharacterized protein | Down/0.73 |
| H3HE38 | Uncharacterized protein | Down/0.78 |
| D0N5G7 | Trifunctional enzyme subunit alpha, putative | Down/0.63 |
| W2PC82 | Uncharacterized protein | Down/0.48 |
| D0NZS4 | Choline/Carnitine O-acyltransferase, putative | Down/0.69 |
| G4Z7L2 | Putative uncharacterized protein | Down/0.62 |
| H3H1D7 | Uncharacterized protein | Down/0.53 |
| A0A0W8DSY3 | Uncharacterized protein | Down/0.75 |
| H3GIW4 | Uncharacterized protein | Down/0.71 |
| H3GS62 | Uncharacterized protein | Down/0.50 |
| A0A0W8D3X9 | Pyruvate dehydrogenase | Down/0.65 |
| A0A0W8C9D8 | Niemann-Pick C1 protein | Down/0.58 |
| H3GIG8 | Uncharacterized protein | Down/0.69 |
| D0MZR7 | Alpha-soluble NSF attachment protein, putative | Down/0.81 |
| G4ZEY9 | Putative uncharacterized protein | Down/0.67 |
| H3GP70 | Uncharacterized protein | Down/0.70 |
| A0A081AFV2 | Uncharacterized protein | Down/0.72 |
| A0A0W8DTC2 | Uncharacterized protein | Down/0.70 |
| G4ZI83 | Peptide deformylase | Down/0.78 |
| W2JIQ1 | Uncharacterized protein | Down/0.53 |
| G4Z589 | Putative uncharacterized protein | Down/0.80 |
| G4ZN62 | Putative uncharacterized protein | Down/0.82 |
| A0A0W8BK50 | Amino Acid/Auxin Permease (AAAP) Family | Down/0.59 |
| D0MYP0 | Putative uncharacterized protein | Down/0.70 |
| D0MS08 | Phospholipase D, Pi-PLD-like-1 | Down/0.72 |
| A0A0W8C4N9 | Phospholipase D alpha 1 | Down/0.68 |
| W2HE96 | Uncharacterized protein | Down/0.58 |
| H3GAX7 | Uncharacterized protein | Down/0.46 |
| A0A0W8D7T5 | Alcohol dehydrogenase 3 | Down/0.66 |
| D0P1W1 | 40S ribosomal protein S14 | Down/0.79 |
| H3G8X2 | Obg-like ATPase 1 | Down/0.82 |
| G5ABJ2 | Putative uncharacterized protein | Down/0.56 |
| G5A628 | Putative uncharacterized protein | Down/0.66 |
| D0NXN9 | Putative uncharacterized protein | Down/0.76 |
| D0NIU2 | HECT E3 ubiquitin ligase, putative | Down/0.71 |
| H3H057 | Uncharacterized protein | Down/0.64 |
| G4YPN6 | Putative uncharacterized protein | Down/0.81 |
| A0A0W8BVH2 | Aldehyde dehydrogenase | Down/0.83 |
| D0N752 | Putative uncharacterized protein | Down/0.77 |
| D0MQG3 | 3-oxoacyl-[acyl-carrier-protein] synthase, mitochondrial | Down/0.69 |
| A0A0W8DGB4 | ATP-dependent RNA helicase DBP2 | Down/0.61 |
| D0MR07 | Nucleoredoxin, putative | Down/0.65 |
| H3HCZ5 | Uncharacterized protein | Down/0.64 |
| A0A080ZKT5 | Acetyl-CoA carboxylase, biotin carboxylase subunit | Down/0.76 |
| H3HCJ6 | Uncharacterized protein | Down/0.79 |
| D0NR26 | Dihydrolipoyl dehydrogenase | Down/0.72 |
| H3GH83 | Uncharacterized protein | Down/0.74 |
| A0A0W8CSZ0 | Uncharacterized protein | Down/0.73 |
| G5A1F5 | Succinate--CoA ligase [ADP-forming] subunit beta, mitochondrial | Down/0.66 |
| H3HAQ3 | RNA helicase | Down/0.69 |
| A0A0W8AY68 | Malate synthase | Down/0.46 |
| A0A0W8BV17 | Uncharacterized protein | Down/0.78 |
| V9ERS5 | Uncharacterized protein | Down/0.59 |
| G4YUN9 | Putative uncharacterized protein | Down/0.80 |
| W2LXT4 | Uncharacterized protein | Down/0.74 |
| H3GD17 | Uncharacterized protein | Down/0.79 |
| D0N1Y4 | Putative uncharacterized protein | Down/0.66 |
| D0N897 | Glutamate synthase, putative | Down/0.63 |
| W2I9Z4 | Uncharacterized protein | Down/0.72 |
| A0A0W8DAM1 | Uncharacterized protein | Down/0.81 |
| D0ND43 | Putative uncharacterized protein | Down/0.77 |
| D0NEQ1 | Prolyl-tRNA synthetase, putative | Down/0.69 |
| G5ADR0 | Putative uncharacterized protein | Down/0.72 |
| D0NAT6 | Putative uncharacterized protein | Down/0.56 |
| D0NLB4 | Mitochondrial Carrier (MC) Family | Down/0.70 |
| H3GMG0 | Purple acid phosphatase | Down/0.53 |
| D0NMG0 | Putative uncharacterized protein | Down/0.69 |
| H3G6Q6 | Uncharacterized protein | Down/0.58 |
| G5AEY9 | Putative uncharacterized protein | Down/0.71 |
| A0A0W8CQB0 | Spore wall maturation protein DIT1 | Down/0.55 |
| H3H7G0 | Uncharacterized protein | Down/0.77 |
| A0A0W8DAF6 | Ribonucleases P/MRP protein subunit POP1 | Down/0.81 |
| W2RFQ0 | Uncharacterized protein | Down/0.61 |
| G4YIK8 | Putative uncharacterized protein | Down/0.69 |
| W2J5J6 | Uncharacterized protein | Down/0.74 |
| G4YUH7 | Putative uncharacterized protein | Down/0.68 |
| D0NXU5 | tRNA (guanine-N(7)-)-methyltransferase non-catalytic subunit | Down/0.71 |
| W2P2C0 | Uncharacterized protein (Fragment) | Down/0.41 |
| H3HE04 | Uncharacterized protein | Down/0.51 |
| H9XQ43 | Dihydroorotase (Fragment) | Down/0.75 |
| H3GU11 | Uncharacterized protein | Down/0.82 |
| D0N4E7 | Putative uncharacterized protein | Down/0.66 |
| A0A0W8DJC2 | Peptidyl-prolyl cis-trans isomerase | Down/0.72 |
| W2ZKP5 | MIP18 family protein | Down/0.78 |
| D0MXQ7 | Putative uncharacterized protein | Down/0.78 |
| A0A0W8DI99 | Uncharacterized protein | Down/0.75 |
| A0A0W8CAE1 | Uncharacterized protein | Down/0.77 |
| A0A081AP93 | Uncharacterized protein | Down/0.57 |
| G4YGQ1 | Putative uncharacterized protein | Down/0.76 |
| V9FQ64 | Uncharacterized protein | Down/0.78 |
| G4ZE43 | Putative uncharacterized protein | Down/0.77 |
| A0A0W8DKD6 | Inositol 2-dehydrogenase | Down/0.67 |
| A0A0W8CC12 | Golgi-specific brefeldin A-resistance guanine nucleotide exchange factor 1 | Down/0.78 |
| D0NX91 | Choline/Carnitine O-acyltransferase, putative | Down/0.66 |
| G4YUI1 | Putative uncharacterized protein | Down/0.77 |
| D0NDY1 | Acyl-CoA dehydrogenase family member 9, mitochondrial | Down/0.76 |
| W2JSX6 | Protein disulfide-isomerase domain | Down/0.71 |
| A0A081A1X3 | Uncharacterized protein | Down/0.75 |
| G4ZRG7 | Putative uncharacterized protein | Down/0.83 |
| D0MXY8 | Anoctamin-like protein | Down/0.82 |
| H3G840 | Uncharacterized protein | Down/0.73 |
| H3GTV9 | Uncharacterized protein | Down/0.64 |
| D0MRG2 | Plasma membrane ATPase | Down/0.68 |
| D0MSB1 | 3-phosphoinositide-dependent protein kinase, putative | Down/0.79 |
| G5AD00 | Putative uncharacterized protein | Down/0.80 |
| D0MUS0 | 40S ribosomal protein S8 | Down/0.61 |
| V9FMK4 | Kinesin-like protein (Fragment) | Down/0.70 |
| D0N2W0 | L-gulonolactone oxidase, putative | Down/0.73 |
| H3HC47 | Uncharacterized protein | Down/0.40 |
| W2HW46 | Uncharacterized protein | Down/0.74 |
| A0A0W8D741 | ATP-binding Cassette (ABC) Superfamily | Down/0.75 |
| H3GD72 | Uncharacterized protein | Down/0.77 |
| A0A1L1UMB8 | Crinkling and necrosis inducing protein 10 (Fragment) | Down/0.80 |
| D0NS71 | Glutamyl-tRNA(Gln) amidotransferase subunit A, putative | Down/0.59 |
| D0N907 | DEAD/DEAH box RNA helicase, putative | Down/0.76 |
| G4ZTJ9 | Putative uncharacterized protein (Fragment) | Down/0.79 |
| H3G903 | Uncharacterized protein | Down/0.61 |
| H3GNB4 | Uncharacterized protein | Down/0.65 |
| D0N1Q9 | Methylcrotonoyl-CoA carboxylase subunit alpha, putative | Down/0.77 |
| A0A0W8C2S9 | Uncharacterized protein | Down/0.59 |
| P22131 | Actin-1 | Down/0.55 |
| D0NXY8 | Succinate dehydrogenase [ubiquinone] flavoprotein subunit, mitochondrial | Down/0.40 |
| D0NLJ9 | Adenosine kinase | Down/0.40 |
| A0A0W8D3W5 | Vesicle transport protein GOT1A | Down/0.68 |
| W2JVW3 | Uncharacterized protein | Down/0.66 |
| A0A081ARE9 | Uncharacterized protein (Fragment) | Down/0.54 |
| A0A0W8C3K9 | Uncharacterized protein | Down/0.70 |
| A0A0W8CTG0 | Coiled-coil domain-containing protein 37 | Down/0.72 |
| G5A0S8 | Putative uncharacterized protein | Down/0.65 |
| G4YQY4 | Putative uncharacterized protein | Down/0.52 |
| A0A0W8D2C2 | Arabinan endo-1 | Down/0.72 |
| G5A9R7 | Putative uncharacterized protein | Down/0.83 |
| D0N1H6 | Putative uncharacterized protein | Down/0.77 |
| G4YP91 | Putative uncharacterized protein | Down/0.60 |
| H3GAD3 | Uncharacterized protein | Down/0.76 |
| W2H3N1 | Uncharacterized protein | Down/0.64 |
| W2YZU5 | Uncharacterized protein (Fragment) | Down/0.66 |
| H3HE08 | Uncharacterized protein | Down/0.78 |
| G4YUC8 | Putative uncharacterized protein | Down/0.64 |
| W2HLN4 | Uncharacterized protein | Down/0.54 |
| H3HC38 | Uncharacterized protein | Down/0.81 |
| A0A0W8DBX2 | Carboxypeptidase | Down/0.54 |
| W2K1R0 | Uncharacterized protein | Down/0.55 |
| A0A0W8DAI5 | Uncharacterized protein | Down/0.64 |
| D0N7X5 | Serine/threonine-protein phosphatase | Down/0.69 |
| H3HCT3 | Uncharacterized protein | Down/0.74 |
| G4YJB2 | Putative uncharacterized protein | Down/0.62 |
| H3GWJ5 | Uncharacterized protein | Down/0.73 |
| W2MV88 | Homoserine dehydrogenase | Down/0.74 |
| H3GGK3 | Uncharacterized protein | Down/0.74 |
| G4XXU1 | Tubulin beta chain | Down/0.81 |
| V9E826 | Oxoglutarate dehydrogenase (Succinyl-transferring), E1 component, variant 5 | Down/0.62 |
| D0N5Q5 | Tryptophan synthase | Down/0.61 |
| V9FAU9 | Uncharacterized protein | Down/0.75 |
| A0A0W8CP12 | AP-4 complex accessory subunit tepsin | Down/0.77 |
| A0A0W8CM06 | Allantoicase | Down/0.62 |
| A0A080ZPP6 | Uncharacterized protein | Down/0.67 |
| H3G8A5 | Peptidylprolyl isomerase | Down/0.81 |
| H3GHV9 | Structural maintenance of chromosomes protein | Down/0.59 |
| W2NI26 | Uncharacterized protein | Down/0.50 |
| G4ZP07 | Putative uncharacterized protein | Down/0.75 |
| A0A0W8C7P7 | Uncharacterized protein | Down/0.77 |
| A0A0W8C7H2 | Thioredoxin | Down/0.82 |
| H3GQC8 | Uncharacterized protein | Down/0.70 |
| D0NI16 | Transcription initiation factor TFIID subunit, putative | Down/0.78 |
| A0A0W8BVS2 | E3 ubiquitin-protein ligase MARCH8 | Down/0.74 |
| G4YSE0 | Putative uncharacterized protein | Down/0.81 |
| V9FVB3 | Protein disulfide-isomerase domain | Down/0.81 |
| D0NIW2 | Protein-L-isoaspartate O-methyltransferase | Down/0.75 |
| D0NN44 | Bifunctional aspartokinase/homoserine dehydrogenase, putative | Down/0.61 |
| A0A0W8BVF0 | Cathepsin cysteine protease | Down/0.50 |
| G4YTV9 | 40S ribosomal protein S24 | Down/0.78 |
| H3G6H0 | Uncharacterized protein | Down/0.51 |
| D0N3V9 | Casein kinase, putative | Down/0.76 |
| G4Z4X0 | Putative uncharacterized protein | Down/0.73 |
| G4YXU1 | Putative uncharacterized protein | Down/0.83 |
| D0MTF4 | Putative uncharacterized protein | Down/0.77 |
| G5A7D6 | Phosphomannomutase | Down/0.65 |
| G4ZSH8 | Putative uncharacterized protein | Down/0.56 |
| D0MSA8 | Ras family GTPase, putative | Down/0.71 |
| A0A080Z0B0 | Uncharacterized protein | Down/0.80 |
| H3HC28 | Uncharacterized protein | Down/0.68 |
| W2HU53 | Uncharacterized protein | Down/0.79 |
| D0NFZ7 | Putative uncharacterized protein | Down/0.78 |
| A0A080ZCR8 | Uncharacterized protein | Down/0.75 |
| H3G856 | Uncharacterized protein | Down/0.54 |
| A0A0W8DB69 | Serine/threonine-protein kinase drkD | Down/0.72 |
| H3G907 | Carboxypeptidase | Down/0.79 |
| A0A0W8D394 | Uncharacterized protein | Down/0.79 |
| H3GJ24 | Carboxypeptidase | Down/0.64 |
| W2MKM4 | Uncharacterized protein | Down/0.54 |
| D0NRY0 | Nucleolar protein, putative | Down/0.69 |
| A0A0W8DEH0 | Phospho-2-dehydro-3-deoxyheptonate aldolase | Down/0.75 |
| A0A0W8DRH5 | Lysosomal thioesterase PPT2-A | Down/0.70 |
| G4Z4Z0 | Neprilysin CD10, peptidase | Down/0.72 |
| H3GJZ1 | Uncharacterized protein | Down/0.52 |
| H3G735 | Mitogen-activated protein kinase | Down/0.71 |
| A0A0W8DKX4 | Diacylglycerol O-acyltransferase 2 | Down/0.82 |
| D0NM95 | ATP-binding Cassette (ABC) Superfamily | Down/0.67 |
| D0MUG5 | Glyceraldehyde-3-phosphate dehydrogenase | Down/0.83 |
| W2PQ06 | Uncharacterized protein | Down/0.63 |
| G4ZIG9 | Putative uncharacterized protein | Down/0.53 |
| D0N1Q4 | Putative uncharacterized protein | Down/0.79 |
| A0A080ZZ10 | Uncharacterized protein | Down/0.63 |
| A0A0W8DU86 | U5 small nuclear ribonucleoprotein component | Down/0.67 |
| A0A080ZS43 | Uncharacterized protein | Down/0.61 |
| D0MQ88 | Glucan 1,3-beta-glucosidase, putative | Down/0.74 |
| A0A0W8D5V8 | Cell 12A endoglucanase | Down/0.75 |
| H3HCR4 | Uncharacterized protein | Down/0.75 |
| H3GAL0 | Rab GDP dissociation inhibitor | Down/0.81 |
| A0A0W8DMM5 | Ribosome biogenesis protein BOP1 homolog | Down/0.56 |
| G4YY40 | Superoxide dismutase | Down/0.76 |
| D0NJE9 | Anoctamin-like protein | Down/0.80 |
| G4YNR4 | Putative uncharacterized protein | Down/0.39 |
| H3G9D5 | Uncharacterized protein | Down/0.53 |
| A0A0W8BY50 | Uracil catabolism protein 4 | Down/0.76 |
| G4Z7V2 | Putative uncharacterized protein | Down/0.65 |
| H3G7L0 | Serine/threonine-protein phosphatase 2A 55 kDa regulatory subunit B | Down/0.69 |
| H3G5A2 | Uncharacterized protein | Down/0.69 |
| W2KB06 | Uncharacterized protein | Down/0.71 |
| V9F4T6 | AGC/AKT protein kinase | Down/0.78 |
| G4ZGM0 | Putative uncharacterized protein | Down/0.72 |
| G4ZHQ6 | Putative uncharacterized protein (Fragment) | Down/0.71 |
| A0A0W8C6R2 | Uncharacterized protein | Down/0.83 |
| D0N4L1 | Putative uncharacterized protein | Down/0.70 |
| D0NSP5 | Putative uncharacterized protein | Down/0.61 |
| A0A0W8D5P8 | ABC transporter G family member 31 | Down/0.65 |
| A0A0W8DAQ0 | Vesicle transport protein | Down/0.80 |
| A0A0H3TYP5 | Translation elongation factor 1-alpha (Fragment) | Down/0.75 |
| H3GB46 | Uncharacterized protein | Down/0.70 |
| W2G1E8 | Uncharacterized protein | Down/0.82 |
| H3G5Z7 | Uncharacterized protein | Down/0.74 |
| H3GNM2 | Uncharacterized protein | Down/0.62 |
| H3G8B1 | Uncharacterized protein | Down/0.78 |
| H3GJ13 | 1,3-beta-glucanosyltransferase | Down/0.81 |
| A0A081AAP2 | Uncharacterized protein | Down/0.69 |
| H3GA35 | Uncharacterized protein | Down/0.72 |
| G5A933 | Putative uncharacterized protein | Down/0.72 |
| D0NE75 | Putative uncharacterized protein | Down/0.63 |
| G4Z715 | Putative uncharacterized protein | Down/0.59 |
| W2LPQ5 | Uncharacterized protein | Down/0.77 |
| H3G8Z9 | Uncharacterized protein | Down/0.82 |
| I1TGZ1 | Arp2/3 complex 34 kDa subunit (Fragment) | Down/0.83 |
| D0NEK9 | Mitochondrial Carrier (MC) Family | Down/0.66 |
| D0MSX6 | Putative uncharacterized protein | Down/0.70 |
| D0N457 | Putative uncharacterized protein | Down/0.71 |
| H3H5G1 | Uncharacterized protein | Down/0.79 |
| G4YH41 | Putative uncharacterized protein | Down/0.66 |
| W2MVT8 | Uncharacterized protein (Fragment) | Down/0.81 |
| W2Q2H4 | Uncharacterized protein | Down/0.67 |
| G4YVW4 | Nitrilase/cyanide hydratase and apolipo protein N-acyltransferase-like protein | Down/0.46 |
| A0A0W8DAC4 | Ammonium Transporter (Amt) Family | Down/0.57 |
| D0NXA2 | Pentafunctional AROM polypeptide | Down/0.82 |
| H3GB63 | Acetyl-coenzyme A synthetase | Down/0.68 |
| D0MZ50 | Protein kinase | Down/0.76 |
| G4ZVE7 | Nucleoside diphosphate kinase | Down/0.62 |
| A0A081AC78 | Uncharacterized protein | Down/0.67 |
| D0N7H7 | 3-ketoacyl-CoA thiolase, mitochondrial | Down/0.74 |
| A0A0W8BW61 | WASH complex subunit FAM21 | Down/0.61 |
| G4YEI1 | Putative uncharacterized protein | Down/0.74 |
| H3G9M7 | Coatomer subunit alpha | Down/0.75 |
| H3GW60 | Uncharacterized protein | Down/0.74 |
| A0A0W8DT93 | Uncharacterized protein | Down/0.76 |
| H3GBB6 | Uncharacterized protein | Down/0.80 |
| D0NMU8 | Putative uncharacterized protein | Down/0.73 |
| H3HEG2 | Casein kinase II subunit beta | Down/0.59 |
| H3G7T4 | Uncharacterized protein | Down/0.82 |
| D0N2I2 | Alcohol dehydrogenase, putative | Down/0.65 |
| H3GDL5 | Uncharacterized protein | Down/0.82 |
| D0NC41 | Putative uncharacterized protein | Down/0.83 |
| G4YR88 | Putative uncharacterized protein | Down/0.51 |
| D0MSF3 | Cell division protease ftsH | Down/0.62 |
| G4YQ84 | Putative uncharacterized protein | Down/0.55 |
| D0N327 | 3-hydroxyacyl-CoA dehydrogenase, putative | Down/0.67 |
| A0A0W8D104 | ATP-binding Cassette (ABC) Superfamily | Down/0.65 |
| W2GDL7 | Uncharacterized protein (Fragment) | Down/0.78 |
| D0NHM7 | Putative uncharacterized protein | Down/0.77 |
| E5D6U5 | Translation elongation factor 1 alpha (Fragment) | Down/0.80 |
| A0A0W8BZE4 | Regulator of microtubule dynamics protein 1 | Down/0.79 |
| G4ZKD3 | Plasma membrane ATPase | Down/0.82 |
| A0A0W8D1R1 | Uncharacterized protein | Down/0.79 |
| A0A0W8DAD8 | Uncharacterized protein | Down/0.83 |
| H3GS04 | Uncharacterized protein | Down/0.64 |
| D0NDJ3 | Acyl-CoA synthetase short-chain family member, putative | Down/0.62 |
| G4Z1Y9 | NADPH:adrenodoxin oxidoreductase, mitochondrial | Down/0.77 |
| D0MTU6 | Hydroxyacylglutathione hydrolase, putative | Down/0.69 |
| W2IRR1 | Uncharacterized protein | Down/0.76 |
| W2N4U3 | Uncharacterized protein | Down/0.67 |
| G4YH51 | Putative uncharacterized protein | Down/0.72 |
| D0N3N1 | Actin-like protein | Down/0.77 |
| A0A0W8CT07 | Uncharacterized protein | Down/0.74 |
| G4YPP5 | Putative uncharacterized protein (Fragment) | Down/0.52 |
| D0N6X5 | Oxidoreductase, putative | Down/0.76 |
| A0A0W8C0M9 | HD domain-containing protein 2 | Down/0.83 |
| H3G7S4 | Uncharacterized protein | Down/0.63 |
| W2KRM5 | Uncharacterized protein | Down/0.80 |
| A0A081ANI6 | Phosphoglycerate mutase | Down/0.75 |
| A0A080ZPL2 | Uncharacterized protein | Down/0.82 |
| A0A0W8CZP7 | Uncharacterized protein | Down/0.80 |
| H3G9I8 | Uncharacterized protein | Down/0.49 |
| G4YQR6 | Putative uncharacterized protein | Down/0.76 |
| H3H5Y2 | Uncharacterized protein | Down/0.77 |
| A0A0W8CI83 | Gamma-glutamyl hydrolase A | Down/0.72 |
| W2HFG3 | Uncharacterized protein | Down/0.68 |
| G4ZPR5 | Putative uncharacterized protein (Fragment) | Down/0.82 |
| D0MRW0 | Putative uncharacterized protein | Down/0.82 |
| D0MVQ9 | Inorganic phosphate transporter, putative | Down/0.75 |
| A0A0W8D1A5 | E3 ubiquitin-protein ligase HERC1 | Down/0.61 |
| A0A0W8C3L2 | Neutral alpha-glucosidase AB | Down/0.82 |
| A0A0W8D0Z2 | Uncharacterized protein | Down/0.78 |
| D0NSS5 | Putative uncharacterized protein | Down/0.59 |
| D0N1U4 | Arginine biosynthesis bifunctional protein ArgJ, mitochondrial | Down/0.72 |
| D0N597 | H-or Na-translocating F-type, V-type and A-type ATPase (F-ATPase) Superfamily | Down/0.81 |
| A0A0W8D9U3 | Uncharacterized protein | Down/0.75 |
| D0NFW3 | Putative uncharacterized protein | Down/0.68 |
| H3GAJ9 | DNA helicase | Down/0.72 |
| D0MY05 | Protein transporter Sec31A, putative | Down/0.78 |
| A0A0W8DQ56 | Uncharacterized protein | Down/0.77 |
| A0A0W8CQL9 | Cortactin-binding protein 2 | Down/0.55 |
| H3GAB1 | Aldehyde dehydrogenase | Down/0.73 |
| H3GAK3 | Acetyl-coenzyme A synthetase | Down/0.62 |
| A0A0W8DQB9 | Uncharacterized protein | Down/0.76 |
| A0A0W8D976 | Uncharacterized protein | Down/0.81 |
| D0N778 | Putative uncharacterized protein | Down/0.66 |
| H3GZZ8 | Uncharacterized protein | Down/0.75 |
| A0A0W8DV18 | Recoverin family protein | Down/0.74 |
| A0A140HDJ0 | Translation elongation factor 1-alpha (Fragment) | Down/0.82 |
| G4YYP3 | Putative uncharacterized protein | Down/0.68 |
| A0A0W8DU44 | Uncharacterized protein | Down/0.82 |
| D0NWT8 | Putative uncharacterized protein | Down/0.73 |
| W2J1G2 | Uncharacterized protein (Fragment) | Down/0.79 |
| A0A0W8D2L4 | Calcium-activated potassium channel subunit alpha-1 | Down/0.72 |
| A0A0W8CFB5 | Villin-1 | Down/0.64 |
| H5ZW91 | 60S ribosomal protein L10 (Fragment) | Down/0.73 |
| A0A0W8DQF5 | DENN domain-containing protein 5B | Down/0.67 |
| D0NG33 | Putative uncharacterized protein | Down/0.62 |
| G4ZL87 | Putative uncharacterized protein | Down/0.76 |
| W2NE46 | Uncharacterized protein | Down/0.77 |
| G5AFM5 | Kinase | Down/0.80 |
| D0N7E2 | Carboxypeptidase | Down/0.72 |
| Q8H731 | Glutamine synthetase | Down/0.64 |
| D0NY60 | Protein transporter Sec61 subunit alpha, putative | Down/0.78 |
| D0N5N5 | Putative uncharacterized protein | Down/0.72 |
| A7XJT1 | Elongation factor 1-alpha (Fragment) | Down/0.83 |
| A0A0W8DIA4 | Lysosomal beta glucosidase | Down/0.67 |
| W2GVZ2 | Uncharacterized protein (Fragment) | Down/0.71 |
| Q8S316 | Putative endo-1,3-beta-glucanase | Down/0.82 |
| A0A0W8CTB3 | Uncharacterized protein | Down/0.70 |
| H3G6B7 | Uncharacterized protein | Down/0.77 |
| G4Z811 | Putative uncharacterized protein | Down/0.79 |
| A0A0W8BQ60 | Serine/threonine-protein kinase | Down/0.66 |
| D0NV17 | Aldehyde dehydrogenase, putative | Down/0.67 |
| H3GP63 | Uncharacterized protein | Down/0.67 |
| A0A0W8C8A2 | Uncharacterized protein | Down/0.78 |
| G4ZU70 | Putative uncharacterized protein (Fragment) | Down/0.61 |
| D0N7P0 | Mitogen-activated protein kinase (Fragment) | Down/0.80 |
| D0NS69 | Calpain-like protein | Down/0.70 |
| D0N473 | Mitogen-activated protein kinase organizer, putative | Down/0.75 |
| H3H4T9 | Uncharacterized protein | Down/0.79 |
| D0NG21 | T-complex protein 1 subunit eta | Down/0.70 |
| A0A0W8DH06 | Sec14 cytosolic factor | Down/0.83 |
| H3GPE6 | Uncharacterized protein | Down/0.82 |
| D0N554 | Long-chain-fatty-acid-CoA ligase, putative | Down/0.77 |
| D0MT22 | Phosphoserine phosphatase | Down/0.77 |
| A0A080Z9F0 | Uncharacterized protein | Down/0.77 |
| D0NLK9 | Putative uncharacterized protein | Down/0.78 |
| A0A0W8CG13 | Phosphatase 2C and cyclic nucleotide-binding/kinase domain-containing protein | Down/0.64 |
| V9EPW4 | Uncharacterized protein | Down/0.63 |
| D0NCV5 | Putative uncharacterized protein | Down/0.64 |
| W2WZT4 | Uncharacterized protein (Fragment) | Down/0.79 |
| A0A0W8CUB7 | Uncharacterized protein | Down/0.77 |
| H3G853 | Uncharacterized protein | Down/0.73 |
| A0A0W8DFM9 | Uncharacterized protein | Down/0.78 |
| H3GH46 | Uncharacterized protein | Down/0.59 |
| H3GFE9 | Uncharacterized protein | Down/0.81 |
| A0A080Z0S4 | TKL protein kinase | Down/0.61 |
| B8Y7V8 | Thioredoxin peroxidase (Fragment) | Down/0.70 |
| H3GH69 | Uncharacterized protein | Down/0.82 |
| H3HAC5 | Uncharacterized protein | Down/0.46 |
| G4YUN7 | Putative uncharacterized protein | Down/0.72 |
| A0A080Z2K7 | Beta-galactosidase | Down/0.57 |
| A0A0W8CX17 | Isovaleryl-CoA dehydrogenase | Down/0.63 |
| H3GZU7 | Uncharacterized protein | Down/0.83 |
| G5AET0 | Putative uncharacterized protein | Down/0.77 |
| H3GPG0 | Uncharacterized protein | Down/0.65 |
| A0A0W8DC41 | Uncharacterized protein | Down/0.73 |
| G4YEP9 | Putative uncharacterized protein | Down/0.79 |
| G4YN79 | Putative uncharacterized protein | Down/0.82 |
| D0NEC7 | Ubiquitin-specific protease, putative | Down/0.79 |
| A4LAC4 | Actin (Fragment) | Down/0.67 |
| A0A0K2SS21 | Translation elongation factor-1 alpha (Fragment) | Down/0.58 |
| G4YZI4 | Putative uncharacterized protein | Down/0.59 |
| A0A0W8DGR4 | Membralin | Down/0.68 |
| D0P0P2 | Protein kinase, putative | Down/0.77 |
| W2WJF5 | TKL protein kinase | Down/0.65 |
| A0A0W8DLM6 | ADP-ribosylation factor | Down/0.82 |
| H3GCS4 | Uncharacterized protein | Down/0.64 |
| G4ZDA7 | Carboxypeptidase | Down/0.59 |
| G4YXX2 | Putative uncharacterized protein (Fragment) | Down/0.70 |
| A0A0W8CRL3 | Uncharacterized protein | Down/0.71 |
| A0A0W8DPY5 | E3 ubiquitin-protein ligase | Down/0.78 |
| A0A0W8DH13 | Adenosylcobalamin-dependent ribonucleoside-triphosphate reductase | Down/0.63 |
| W2LDZ2 | Uncharacterized protein (Fragment) | Down/0.82 |
| G4YWE4 | Putative uncharacterized protein | Down/0.79 |
| H3HD16 | Uncharacterized protein | Down/0.82 |
| D0N4E3 | 60S ribosomal protein L8, putative | Down/0.83 |
| D0NJQ8 | Putative uncharacterized protein | Down/0.82 |
| D0N4V0 | HECT E3 ubiquitin ligase, putative | Down/0.81 |
| G5A5F6 | Putative uncharacterized protein | Down/0.73 |
| H3GE69 | Uncharacterized protein | Down/0.76 |
| D0MQK8 | Metallophosphoesterase 1, putative | Down/0.65 |
| A0A0W8DKM0 | Uncharacterized protein | Down/0.79 |
| H3G995 | Purine nucleoside phosphorylase | Down/0.77 |
| G4Z8N5 | Putative uncharacterized protein | Down/0.62 |
| D0N1G4 | Formin-homology 2 domain-containing protein | Down/0.64 |
| G4ZK78 | Putative uncharacterized protein | Down/0.65 |
| H3H1E8 | Uncharacterized protein | Down/0.81 |
| G4Z8H6 | Putative uncharacterized protein | Down/0.76 |
| H3GDN9 | Uncharacterized protein | Down/0.69 |
| G5A3M0 | Putative uncharacterized protein | Down/0.77 |
| H3H444 | Uncharacterized protein | Down/0.77 |
| A0A0W8DW29 | Syntaxin-31 | Down/0.77 |
| H3HD85 | Uncharacterized protein | Down/0.77 |
| D0N1M9 | Glucan 1,3-beta-glucosidase, putative | Down/0.62 |
| D0NFX7 | Glycogen Synthase Kinase 3 beta | Down/0.82 |
| H3GFJ2 | Uncharacterized protein | Down/0.83 |
| H3GA59 | Uncharacterized protein | Down/0.83 |
| A0A0W8D526 | Uncharacterized protein | Down/0.60 |
| H3GBY0 | Uncharacterized protein OS=Phytophthora ramorum PE=3 SV=1 - [H3GBY0_PHYRM] | Down/0.78 |
| D0P1T9 | Nicotinate-nucleotide pyrophosphorylase [carboxylating] | Down/0.75 |
| G4Z8Z5 | Putative uncharacterized protein | Down/0.83 |
| H3G513 | Uncharacterized protein | Down/0.82 |
| H3G7I8 | Uncharacterized protein | Down/0.69 |
| G4YED3 | Phospholipase D-like protein | Down/0.78 |
| G4ZWK9 | Putative uncharacterized protein | Down/0.77 |
| A0A0W8CI70 | 40S ribosomal protein S2 | Down/0.73 |
| G4YIN2 | Putative uncharacterized protein | Down/0.51 |
| H3G603 | Uncharacterized protein | Down/0.67 |
| A0A0W8CHZ4 | Uncharacterized protein | Down/0.83 |
| W2NSZ9 | Uncharacterized protein | Down/0.82 |
| V9E850 | Uncharacterized protein | Down/0.83 |
| D0NIG7 | Putative uncharacterized protein | Down/0.74 |
| H3G948 | Superoxide dismutase | Down/0.73 |
| H3GAW2 | Mitochondrial pyruvate carrier | Down/0.82 |
| W2WHT9 | Proteasome endopeptidase complex | Down/0.79 |
| A0A0W8DVB8 | Uncharacterized protein | Down/0.70 |
| A0A0W8CNC1 | RNA exonuclease 4 | Down/0.81 |
| G5A7L2 | Putative uncharacterized protein | Down/0.68 |
| A0A0W8BV53 | Beta-glucosidase 42 | Down/0.65 |
| G4YR43 | Enoyl-CoA hydratase (Fragment) | Down/0.82 |
| G4YM53 | Putative uncharacterized protein | Down/0.78 |
| G4YJV5 | Putative uncharacterized protein (Fragment) | Down/0.69 |
| A0A0W8DEI5 | Uncharacterized protein | Down/0.78 |
