# Supplementary figures and images for: Cinnamaldehyde inhibits the growth of Phytophthora capsici through disturbing metabolic homoeostasis
Source: PeerJ. 2021 Apr 30;9:e11339. doi: 10.7717/peerj.11339 (PMC8092109; doi:10.7717/peerj.11339)

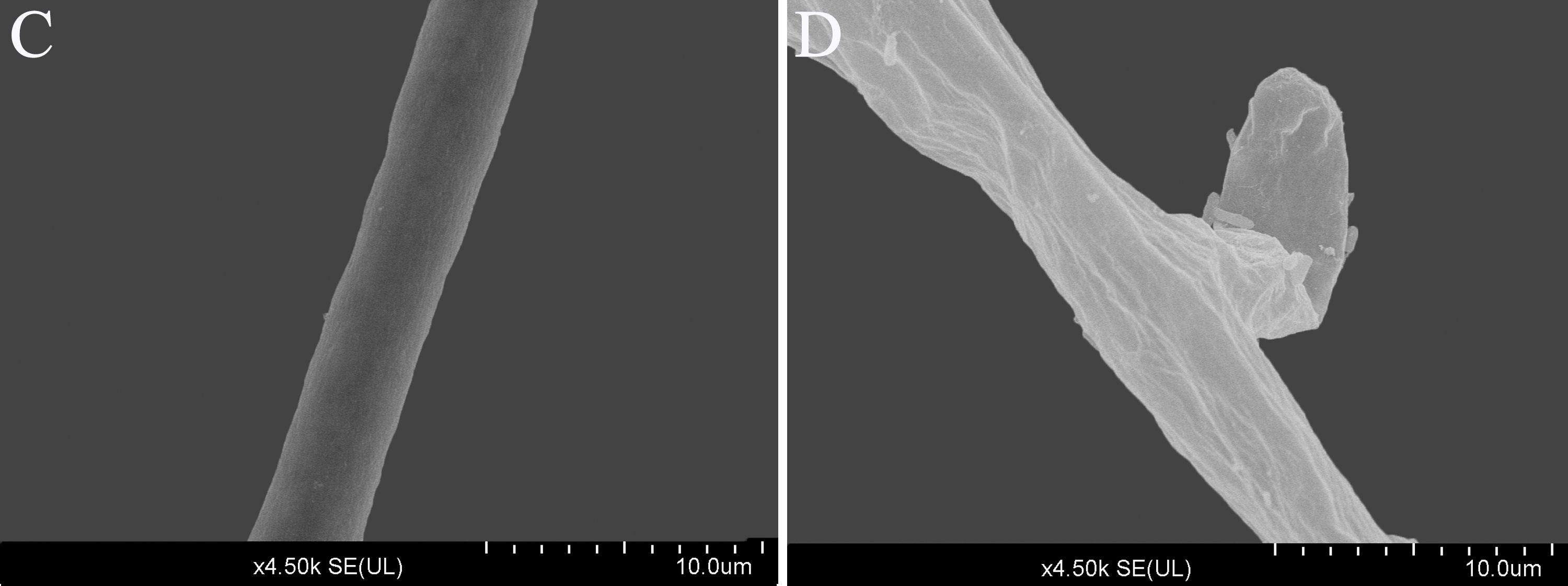

Supplement: Supplemental Information 3 — (C) Mycelium of untreated culture (solvent control) treated for 3 days; (D) mycelium ofP. capsici treated with CA at 140 mg/L for 3 days. [file peerj-09-11339-s003.jpg]
